# Loss of Endothelial Annexin A1 Aggravates Inflammation‐Induched Vascular Aging

**DOI:** 10.1002/advs.202307040

**Published:** 2024-02-15

**Authors:** Qinyi You, Yilang Ke, Xiaofeng Chen, Wanhong Yan, Dang Li, Lu Chen, Run Wang, Jie Yu, Huashan Hong

**Affiliations:** ^1^ Department of Geriatrics Fujian Medical University Union Hospital Fujian Key Laboratory of Vascular Aging (Fujian Medical University) Fujian Institute of Geriatrics Department of Cardiology Fujian Heart Disease Center Fujian Clinical Research Center for Vascular and Brain Aging Fuzhou Fujian 350001 China

**Keywords:** annexin A1, endothelial cell, inflammaging, vascular aging

## Abstract

Chronic inflammation is increasingly considered as the most important component of vascular aging, contributing to the progression of age‐related cardiovascular diseases. To delay the process of vascular aging, anti‐inflammation may be an effective measure. The anti‐inflammatory factor annexin A1 (ANXA1) is shown to participate in several age‐related diseases; however, its function during vascular aging remains unclear. Here, an ANXA1 knockout (ANXA1^−/−^) and an endothelial cell‐specific ANXA1 deletion mouse (ANXA1^△EC^) model are used to investigate the role of ANXA1 in vascular aging. ANXA1 depletion exacerbates vascular remodeling and dysfunction while upregulates age‐ and inflammation‐related protein expression. Conversely, Ac2‐26 (a mimetic peptide of ANXA1) supplementation reverses this phenomenon. Furthermore, long‐term tumor necrosis factor‐alpha (TNF‐α) induction of human umbilical vein endothelial cells (HUVECs) increases cell senescence. Finally, the senescence‐associated secretory phenotype and senescence‐related protein expression, rates of senescence‐β‐galactosidase positivity, cell cycle arrest, cell migration, and tube formation ability are observed in both ANXA1‐knockdown HUVECs and overexpressed ANXA1‐TNF‐α induced senescent HUVECs. They also explore the impact of formyl peptide receptor 2 (a receptor of ANXA1) in an ANXA1 overexpression inflammatory model. These data provide compelling evidence that age‐related inflammation in arteries contributes to senescent endothelial cells that promote vascular aging.

## Graphical abstract

This study investigates the impact of ANXA1 deficiency in both human subjects and mice with endothelial‐specific ANXA1 knockout. The results demonstrate a significant negative correlation between ANXA1 levels and vascular aging. Loss of ANXA1 promotes vascular aging by deteriorating vascular structure and function through chronic inflammation, senescence‐associated secretory phenotype, and arrest of the endothelial cell cycle.

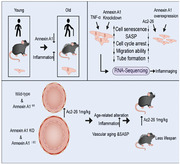

## 1 Introduction

Aging is a major risk factor for cardiovascular diseases (CVDs) such as atherosclerosis, heart failure, and hypertension.^[^ ^1^ ^]^ A healthy aging process depends on the maintenance of a properly functioning vascular system; that is, vascular aging is not only the common pathogenesis of various chronic diseases and contributes physiologically and pathologically to aging at both the cellular and organ levels but also constitutes the onset of organismal aging.^[^ ^2^, ^3^ ^]^ Numerous studies have proposed probable mechanisms, such as oxidative stress, chronic inflammation, mitochondrial dysfunction, and cellular senescence, that occur during vascular aging.^[^ ^4^ ^]^ Our previous study showed that the gut flora‐dependent metabolite trimethylamine‐N‐oxide accelerated vascular aging and endothelial senescence through oxidative stress.^[^ ^5^ ^]^ This finding suggested that during vascular aging, mutual promotive effects of oxidative stress and inflammation result in impairment of arterial function by which inflammatory cytokines exacerbate O_2_^−^ production, and in turn, reactive oxygen species (ROS) stimulate nuclear factor kappa‐B (NF‐κB), the core transcription factor of inflammatory pathways, to induce inflammatory cytokine accumulation.^[^ ^6^ ^]^ The state of low‐grade, long‐term sterile inflammation is referred to as inflammaging,^[^ ^7^ ^]^ and it can lead to structural and functional alterations in large vessels in humans.^[^ ^8^ ^]^ Although the mechanisms underlying vascular aging are incompletely understood, chronic inflammation appears to be the most likely process involved. This finding suggests that anti‐inflammatory molecules can alleviate aging‐related chronic low‐grade inflammation.^[^ ^9^ ^]^

The role of annexin A1 (ANXA1), an anti‐inflammatory regulator as a glucocorticoid‐inducible protein in the tumor formation process has been fully explored; however, ANXA1 was also discovered to be correlated with atherosclerosis by suppressing neutrophil chemotaxis and promoting proinflammatory cytokine resolution.^[^ ^10^, ^11^, ^12^ ^]^ ANXA1 can reduce platelet activation and aggregation, exerting protective effects against thrombosis and inflammation.^[^ ^13^ ^]^ In addition, the lack of ANXA1 has been verified to induce phenotypic switching in vascular smooth muscle cells and to exacerbate acute aortic dissection via^[^ ^14^ ^]^ the JUNB/myosin light chain 9 pathway. ANXA1 can reduce leukocyte adhesion to endothelial cells and have microvascular‐protective effects in the context of metabolic syndrome.^[^ ^15^ ^]^ Collectively, this evidence suggests that ANXA1 might have therapeutic potential in cardiovascular disorders. Moreover, the impact of ANXA1 in other age‐related diseases, such as neurodegenerative diseases^[^ ^16^ ^]^ and diabetes mellitus,^[^ ^17^ ^]^ indicates that ANXA1 might affect the aging process. ANXA1 deficiency was proven to lead to remodeling of the mesenteric vasculature in an insulin‐resistant model,^[^ ^18^ ^]^ demonstrating that ANXA1 may play a vasoprotective role by reducing vascular injury in the context of age‐related diseases. However, the role of ANXA1 in vascular aging remains to be elucidated.

Endothelial cells are an important component of vascular walls and function in processes such as angiogenesis and blood pressure regulation. Endothelial dysfunction accompanied by endothelial senescence results in abnormal blood flow and increased inflammatory factor adhesion, leading to a common vascular aging process.^[^ ^19^ ^]^ Our previous studies have already shown the importance of endothelial function in vascular aging;^[^ ^5^, ^20^ ^]^ therefore, further investigations of endothelial cell senescence are crucial for understanding vascular aging.

In the present study, we compared ANXA1 levels in the serum of humans and mice of different ages to determine the correlation between ANXA1 and aging. We also investigated the alterations in mouse aortic structure and aortic function in ANXA1 knockout (KO) mice in terms of age dynamics and found that these alterations could be rescued by an ANXA1 mimetic peptide named Ac2‐26 and the anti‐inflammatory function of ANXA1 can affect vascular remodeling and vascular stiffness. Aortic structure and function were also investigated in mice with endothelial cell‐specific ANXA1 deletion (ANXA1^△EC^). Furthermore, we observed cell senescence in human umbilical vein endothelial cells (HUVECs) with ANXA1 knockdown and found that senescence was also rescued by Ac2‐26 treatment or ANXA1 overexpression, thus identifying a novel target to prevent or delay vascular aging.

## 2 Results

### 2.1 The ANXA1 Level Correlates Negatively with Age in Healthy Adults

**Figure 1:**
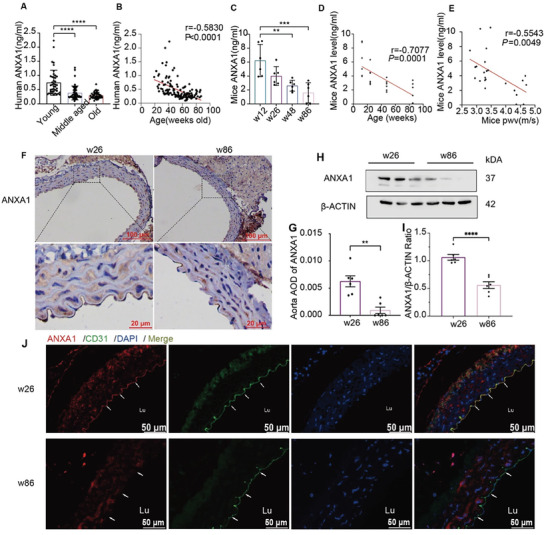
Age‐associated decreases in ANXA1 levels in both humans and mice. A) The level of ANXA1 in serum collected from elderly people (>65 years old, *n* = 43) was significantly lower than that in serum collected from young people (18–44 years old, *n* = 42) or middle‐aged people (45–64 years old, *n* = 48). B) The serum ANXA1 level was negatively correlated with age in humans (*n* = 133). C) The serum level of ANXA1 in mice (*n* = 6 mice per group) was significantly decreased with increasing age. D,E) The serum ANXA1 level was negatively correlated with age and the PWV in mice (*n* = 24). F,G) Representative images of ANXA1 immunohistochemical staining in aortas from 26‐ and 86‐week‐old mice (*n* = 6). Bars = 100 and 20 µm, respectively. The zoomed‐in views showed age‐associated decreased ANXA1 expression in mouse aortas. H,I) Western blotting of mouse aortas showed that ANXA1 expression was decreased in 26‐ and 86‐week‐old mice (*n* = 6). J) Aortas from 26‐ and 86‐week‐old mice were subjected to immunofluorescence staining for ANXA1; representative images are shown (*n* = 6). The arrows show that the decrease in ANXA1 expression was manifested mainly in the vascular endothelium. Bars = 50 µm. Lu, lumen. The data are presented as the means ± SEMs (G,I) and means ± SDs (A,C). The Kruskal–Wallis test (A), one‐way ANOVA (C), and Mann–Whitney *U* test (G,I) were used to compare the data. * *p* < 0.05, ** *p* < 0.01, *** *p* < 0.001, **** *p* < 0.0001; *p* > 0.05 is not indicated. Correlations were determined by Spearman (B) and Pearson correlation analyses (D,E).

To measure ANXA1 levels in human serum from people of different ages who underwent a physical checkup at Fujian Union Hospital between 2019 and 2020, we obtained blood biochemical data from young people (*n* = 42), middle‐aged people (*n* = 48), and elderly people (*n* = 43) (**Figure** **1**A). Elderly people showed significantly lower levels of ANXA1 than young people and middle‐aged people. Correlation analyses demonstrated that the serum ANXA1 level was negatively correlated with age (*r* = −0.5830, *p* < 0.0001) (Figure 1B), suggesting that the ANXA1 level may be related to chronological age and is very likely to be a marker of vascular aging. We also listed the hematological and biochemical parameters of healthy adults (Table S1, Supporting Information) and analyzed the correlations between the serum ANXA1 level and other clinical parameters (Figure S1, Supporting Information). However, no other significant correlations were found, indicating that the serum ANXA1 level could be a unique biomarker for vascular aging.

### 2.2 ANXA1 Expression Decreases with Age in Mice and Correlates with Vascular Aging

The ANXA1 level decreased in mice of increasing ages. As shown in Figure 1C,D, the ANXA1 level gradually decreased in mouse serum from 12‐, 26‐, 48‐, and 86‐week‐old mice and was significantly decreased in 86‐week‐old mice compared to 12‐week‐old mice, which was negatively correlated with age. Furthermore, the negative correlation between ANXA1 expression and arterial stiffness was also evaluated by measurement of the pulse wave velocity (PWV) (Figure 1E). To further investigate the expression of ANXA1 in the aorta, immunohistochemistry (Figure 1F,G), western blotting (Figure 1H,I), and immunofluorescence staining (Figure 1J) were used to quantify ANXA1 expression in mouse aortas. The expression of ANXA1 in the endothelium was obviously decreased in aged mice.

### 2.3 Depletion of ANXA1 Accelerates the Development of Vascular Aging in 68‐Week‐Old Mice

**Figure 2:**
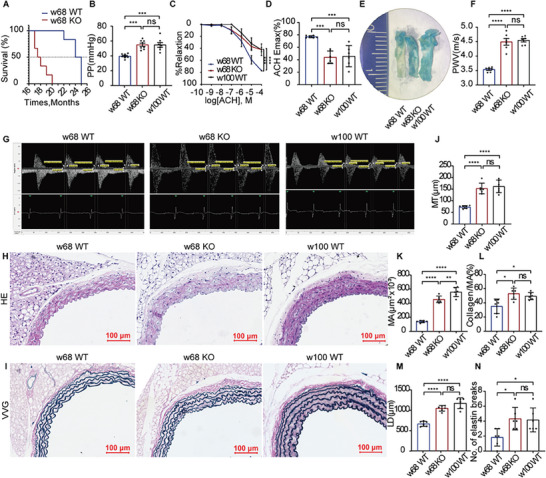
Age‐related alterations in the aorta aggravates in ANXA1 KO mice. A) Reduced lifespan was observed in ANXA1 KO mice, as determined by Kaplan–Meier survival analysis (*n* = 6). B) The PP was elevated in 68‐week‐old (w68) KO mice as well as 100‐week‐old (w100) WT mice compared to w68 WT mice (*n* = 8). C,D) ACH‐induced endothelium‐dependent relaxation of thoracic aortic rings was reduced in both w68 KO mice and w100 WT mice (*n* = 6). E) SA‐β‐gal staining of the aorta showed a larger positive area in w68 KO mice and w100 WT mice (*n* = 6). F,G) The aortic PWV was higher than that in w100 WT mice (*n* = 6). H–N) w68 KO mice and w100 WT mice showed enlargement of the lumen and thickening of the aortic intima and media compared to w68 WT mice. The data also showed increases in collagen deposition and elastin breaks in both w68 KO mice and w100 WT mice. Vascular remodeling indicators, such as the MT, MA, LD, collagen area/MA, and number of elastin breaks, were analyzed by HE staining and VVG staining of mouse aortas. Bars = 100 µm. The data are presented as the means ± SEMs (B,F) and means ± SDs (A,D,J–N). Kaplan–Meier survival analysis (A), one‐way ANOVA followed by the Bonferroni post hoc test (B,D,F,J–N), and multiple repeated measures ANOVA followed by the Bonferroni post hoc test (C) were used for the analyses. *p < 0.05, **p < 0.01, ***p < 0.001, ****p < 0.0001. ns, *p* > 0.05.

We obtained ANXA1 KO mice to explore whether ANXA1 deficiency impairs vascular structure and function (Figure S2, Supporting Information). We used 12‐ and 68‐week‐old mice to investigate vascular impairment with age since KO mice began to die after 68 weeks of age. Naturally, aged C57BL/6J mice at 100 weeks served as the positive control. ANXA1*^−/−^* mice showed a shorter median survival time (months) and lower survival rate than their control littermates (**Figure** **2**A).

To evaluate vascular function, pulse pressure (PP), PWV, and vasomotion were measured. The PP and PWV were significantly elevated in ANXA1*^−/−^* mice compared to WT mice in 68‐week‐old KO mice and 100‐week‐old mice compared to age‐matched control mice (Figure 2B,F,G); however, no significance was observed in mice at 12 weeks old (Figure S3B,D, Supporting Information). Acetylcholine (ACH)‐induced endothelium‐dependent vasodilation was weaker and less sensitive in ANXA1 KO mice and 100‐week‐old mice than in age‐matched control mice (Figure 2C,D and Figure S3E,F, Supporting Information). However, no large differences were observed between wild‐type (WT) and ANXA1 KO mice in body mass, heart rate (HR), sodium nitroprusside (SNP)‐induced endothelium‐independent vasodilatory function, and phenylephrine (PHE)‐induced contractile function (Figures S3A–C,G–J and S4A–H, Supporting Information). Senescence‐associated beta‐galactosidase (SA‐β‐gal) staining demonstrated an increased positive area in the aortic intima in KO mice at 68 weeks and in C57BL/6J mice at 100 weeks compared to that in WT control mice with no significance at 12 weeks. (Figure 2E and Figure S3X, Supporting Information).

Vascular remodeling was evaluated by hematoxylin‐eosin (HE) staining and Verhoeff–Van Gieson (VVG) staining (Figure 2H,I and Figure S3K,L, Supporting Information). Vascular remodeling parameters, such as the medial thickness (MT), lumen diameter (LD), medial area (MA), ratio of the collagen content to the medial area (collagen/MA), and number of elastin breaks in the aorta, were higher in both ANXA1^−/−^ mice at 68 weeks of age and in aged mice than in age‐matched WT mice (Figure 2J–N). However, we did not find any differences in these indicators between WT and KO mice at 12 weeks of age (Figure S3M–R, Supporting Information).

**Figure 3:**
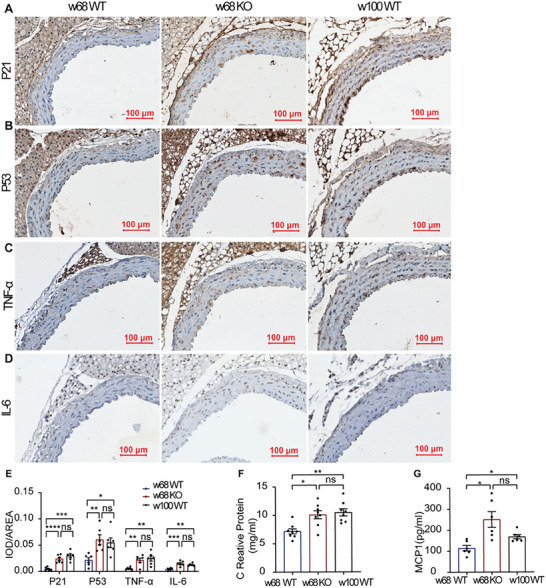
Inflammaging‐related protein elevates in 68‐week‐old ANXA1 KO mice. A–E) w68 KO mice, as well as w100 WT mice, showed higher expression of senescence indicators, such as P21 and P53, and inflammatory cytokines, such as TNF‐α and IL‐6, than control mice. Expression levels were measured by immunohistochemical staining (*n* = 6). Bars = 100 µm. F,G) CRP (*n* = 8) and MCP1 (*n* = 6) levels were elevated in the serum of w68 KO and w100 WT mice compared to those in the serum of w68 WT mice. The data are presented as the means ±SEMs (F,G) and means ± SDs (E). One‐way ANOVA followed by the Bonferroni post hoc test (E–G) was used for the analyses. *p < 0.05, **p < 0.01, ***p < 0.001, ****p < 0.0001. ns, *p* > 0.05.

Moreover, the expression of P53, P21, and senescence‐associated secretory phenotype (SASP) factors, such as tumor necrosis factor‐alpha (TNF‐α) and interleukin (IL)‐6, was increased significantly in both ANXA1*^−/−^* mice at 68 weeks of age and in old mice compared to control mice (**Figure** **3**A–E); nevertheless, only P53 and TNF‐α were significantly elevated in the KO group in 12‐week‐old mice (Figure S3S–W, Supporting Information). The results with the negative control and IgG isotype control are shown in Figure S7I, Supporting Information.

Furthermore, monocyte chemotactic protein‐1 (MCP1) and C reactive protein (CRP) levels were increased in the serum of ANXA1^−/−^ mice at 68 weeks of age and in old (100 weeks) C57BL/6J mice as inflammatory indicators (Figure 3F,G).

Microvascular rarefaction is also a potential indicator of aging, since it is linked to several age‐associated diseases such as diabetes^[^ ^17^ ^]^ and obesity.^[^ ^21^ ^]^ To figure out the alteration of microvascular density in other organs, Skin Masson staining was performed to provide a clear view of subdermal microvascular rarefaction in 68 weeks ANXA1^−/−^ mice (Figure S8A, Supporting Information). Studies^[^ ^22^ ^]^ reported that the switch from brown adipose to white adipose signified age‐associated metabolic diseases. To observe the adipose alteration in KO mice, we performed HE staining of both brown adipose tissue (BAT) and white adipose tissue (WAT) and observed that in old mice, BAT lost their brown‐like phenotype and turned to whitening adipocytes that contained large lipid droplets which also happened in ANXA1^−/−^ mice (Figure S8B,C, Supporting Information). Similarly, white adipose turned out to have larger adipocytes in ANXA1^−/−^ mice and old mice compared to that in the control, which reflected the senescence situation in adipose tissue.

Female mice were also investigated through the survival rate (Figure S6B, Supporting Information), appearance, PP, PWV, and vasomotion (Figure S4A–M, Supporting Information). Vascular remodeling (Figure S4N–U, Supporting Information) was evaluated, and was also found no significant differences with male mice. P53 and P21 protein expression were also upregulated in female w68 KO mice (Figure S5A–D, Supporting Information).

Taken together, the results suggested that depletion of ANXA1 compromised normal vascular tone and led to arterial stiffness and inflammaging in mouse aortas at 68 weeks, a result similar to that observed in naturally aging mice at 100 weeks.

### 2.4 Ac2‐26 Mimetic Peptide Treatment Improves Vascular Aging in Terms of Structure and Function

**Figure 4:**
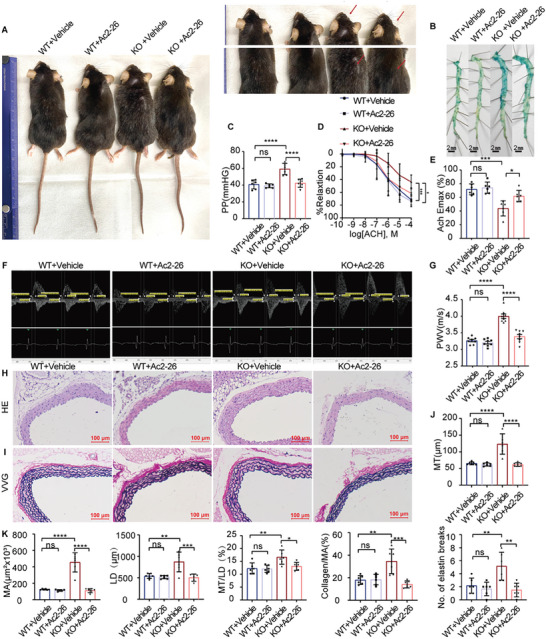
Treatment with Ac2‐26 alleviates the aging process in ANXA1 KO mice. A) Ac2‐26 treatment improved the appearance of ANXA1 KO mice. B) The area of positive SA‐β‐gal staining in the aorta was reduced in ANXA1 KO mice treated with Ac2‐26 (*n* = 6). C) The PP in ANXA1 KO mice was reduced by Ac2‐26 treatment (*n* = 6). D,E) ACH‐induced endothelium‐dependent relaxation of thoracic aortic rings was rescued by Ac2‐26 treatment in KO mice. Maximum relaxation induced by ACH (*n* = 6). F,G) The decreased PWV indicated that the degree of aortic stiffness was decreased with the Ac2‐26 treatment compared to that in the ANXA1 KO group (*n* = 8). H–K) Collagen accumulation and elastin breaks were also improved by Ac2‐26 with HE staining and VVG staining. Enlargement of the lumen, intima‐media thickening, collagen accumulation, and breaks in elastin were improved after Ac2‐26 treatment in KO mice. The data are presented as the means ±SEMs (C,G) and means ±SDs (D,E,J,K). Two‐way ANOVA followed by the Bonferroni post hoc test (C,E,G,J,K) and multiple repeated measures ANOVA followed by the Bonferroni post hoc test (D) were used for the analyses. Bars = 100 µm. *p < 0.05, **p < 0.01, ***p < 0.001, ****p < 0.0001. ns, *p* > 0.05.

To verify whether ANXA1 could provide vascular protection, ANXA1 WT, and KO mice were treated with vehicle (Phosphate‐Buffered saline, PBS) or the ANXA1 mimetic peptide Ac2‐26 (1 mg kg^−1^) for 4 months. Mice that did not receive Ac2‐26 treatment had whitening fur and displayed sparse body and facial fur, while Ac2‐26 treatment effectively attenuated the above effects (**Figure** **4**A). The SA‐β‐gal activity in the mouse aortic tunica intima decreased obviously after Ac2‐26 treatment in KO mice (Figure 4B). Elevation of the PP was observed in KO mice, while the PP decreased significantly after Ac2‐26 treatment (Figure 4C). Ac2‐26 treatment also reversed the increase in PWV in ANXA1 KO mice (Figure 4F,G). Moreover, aortic endothelium‐dependent vasodilation was rescued and the maximum degree of relaxation was increased in the Ac2‐26 treatment group (Figure 4D,E). However, the degrees of PHE‐induced vascular contraction and SNP‐induced endothelium‐independent vasodilation were not significantly different among the four groups (Figure S7J–M, Supporting Information).

**Figure 5:**
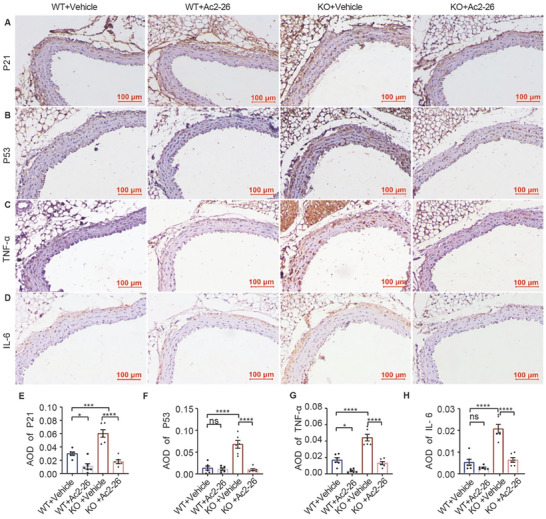
Treatment with Ac2‐26 alleviates inflammaging in ANXA1 KO mice. A–H) The expression of P21 and P53 and SASP factors, such as TNF‐α and IL‐6, was reduced with Ac2‐26 treatment in KO mice, as shown by immunohistochemical staining (*n* = 6). The data are presented as the means ±SDs (E–H). Two‐ way ANOVA followed by the Bonferroni post hoc test (E–H) was used for the analyses. *p < 0.05, **p < 0.01, ***p < 0.001, ****p < 0.0001. ns, *p* > 0.05.

Furthermore, a structural exacerbation was attenuated by Ac2‐26 treatment. Ac2‐26 treatment significantly improved the MT, LD, MA, MT/LD, collagen/MA, and number of elastin breaks in the aorta compared to those in the vehicle treatment group of KO mice (Figure 4H–K). In addition, the expression of P53, P21, and inflammatory cytokines, such as TNF‐α and IL‐6, was significantly decreased in the aortas of mice treated with Ac2‐26 (**Figure** **5**A–H).

### 2.5 Establishment of an Endothelial Cell‐Specific ANXA1 Deletion Mouse (ANXA1^△EC^) Model for Vascular Aging

**Figure 6:**
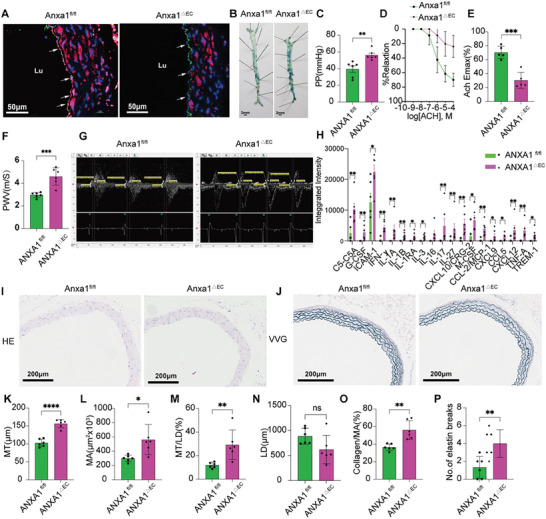
Age‐related alterations and inflammatory factors in the aorta aggravate in ANXA1^△EC^ mice. A) Identification of ANXA1^△EC^ mice aorta with immunofluorescence for ANXA1; representative images are shown (*n* = 6). Green represents CD31 and red represents ANXA1 with blue for the nucleus. Bars = 50 µm. B) SA‐β‐gal staining of the aorta showed a larger positive area in the endothelium of ANXA1^△EC^ mice (*n* = 6). C) The PP was elevated in ANXA1^△EC^ mice compared to ANXA1^fl/fl^ mice (*n* = 6). D,E) ACH‐induced endothelium‐dependent relaxation of thoracic aortic rings was reduced in ANXA1^△EC^ mice (*n* = 6). F,G) The aortic PWV was higher than that in ANXA1^△EC^ mice (*n* = 6). H) The expression of inflammation‐related cytokines, such as Complement component 5a (C5a), Granulocyte colony‐stimulating factor (G‐CSF), Intercellular cell adhesion molecule‐1 (ICAM‐1), Interferon‐γ(IFN‐γ), IL‐1α, IL‐1β, IL‐1ra, IL‐3, IL‐16, IL‐17, IL‐27, C‐X‐C motif chemokine ligand‐10 (CXCL10), Macrophage colony‐stimulating factor (M‐CSF), Monocyte chemoattractant protein‐1 (MCP1), CXCL9, CCL5, CXCL12, TNF‐α, and Human myeloid cell trigger receptor‐1 (TREM‐1) were increased in ANXA1^△EC^ mice group, as shown by proteome profiler mouse cytokine array kit (*n* = 6). I–P) ANXA1^△EC^ mice showed enlargement of the lumen and thickening of the aortic intima and media compared to w68 WT mice. The data also showed increases in collagen deposition and elastin breaks in ANXA1^△EC^ mice. Vascular remodeling indicators, such as the MT, MA, LD, MT/LD, collagen area/MA, and number of elastin breaks, were analyzed by HE staining and VVG staining of mouse aortas. Bars = 200 µm. The data are presented as the means ± SEMs (C,F,H) and means ± SDs (E,F,K–P). Two‐tailed unpaired Student's *t*‐test (C,E,F,H,K–P), and multiple repeated measures ANOVA followed by the Bonferroni post hoc test (D) were used for the analyses. *p < 0.05, **p < 0.01, ***p < 0.001, ****p < 0.0001. ns, *p* > 0.05.

To ascertain whether ANXA1 exerts an antivascular aging function in mice, we validated an endothelial cell‐specific ANXA1 deletion mouse (ANXA1^△EC^) model for age‐related aortic structure and function. Immunohistochemistry (**Figure** **6**A) and mouse tail genotyping (Figure S2C, Supporting Information) were used for the identification of ANXA1^fl/fl^ and ANXA1^△EC^ mice separately. ANXA1^△EC^ mice showed a decreased survival lifetime (Figure S6A, Supporting Information). Senescence‐associated beta‐galactosidase (SA‐β‐gal) staining demonstrated an increased positive area in the aortic intima in ANXA1^△EC^ mice at 68 weeks compared to that in ANXA1^fl/fl^ mice. (Figure 6B). Furthermore, the PP and PWV were significantly higher in the ANXA1^△EC^ mouse group than in the ANXA1^fl/fl^ group at 68 weeks of age, while endothelial‐dependent vasodilation was worse in ANXA1^△EC^ mice (Figure 6C–G). Serum inflammatory factors were quantitatively analyzed by proteomic cytokine microarray in ANXA1^△EC^ and ANXA1^fl/fl^ mice at 68 weeks of age. As shown in Figure 6H, the serum levels of inflammatory factors, such as Complement conponent 5a, Granulocyte colony‐stimulating factor, Intercellular cell adhesion molecular‐1 (ICAM‐1), Interferon‐γ, IL‐1a, IL‐1b, IL‐1ra, IL‐3, IL‐16, IL‐17, IL‐27, C‐X‐C motif chemokine ligand 10 (CXCL10), Macrophage colony‐stimulating factor, Monocyte chemoattractant protein 1 (CCL‐2/MCP1), CXCL9, CCL5, CXCL12, TNF‐α, and Human myeloid cell trigger receptor‐1, in ANXA1^△EC^ were significantly higher than those in the 68‐week‐old ANXA1^fl/f^ group, reflecting that deletion of ANXA1 in the endothelium drives the whole body into an inflammatory‐state. Vascular remodeling was evaluated by HE staining and VVG staining (Figure 6I,J). Vascular remodeling parameters, such as MT, MT/LD, MA, collagen/MA, and the number of elastin breaks in the aorta, were higher in ANXA1^△EC^ mice at 68 weeks of age than in age‐matched ANXA1^fl/fl^ mice (Figure 6K–P), suggesting that ANXA1^△EC^ mice demonstrated age‐related vascular structure. Furthermore, the expression of P53 and P21 was increased significantly in ANXA1^△EC^ mice at 68 weeks of age compared to ANXA1^fl/fl^ mice (Figure S2D–F, Supporting Information).

### 2.6 ANXA1 Expression Decreases During TNF‐α‐Induced HUVEC Senescence and Replicative Senescence

**Figure 7:**
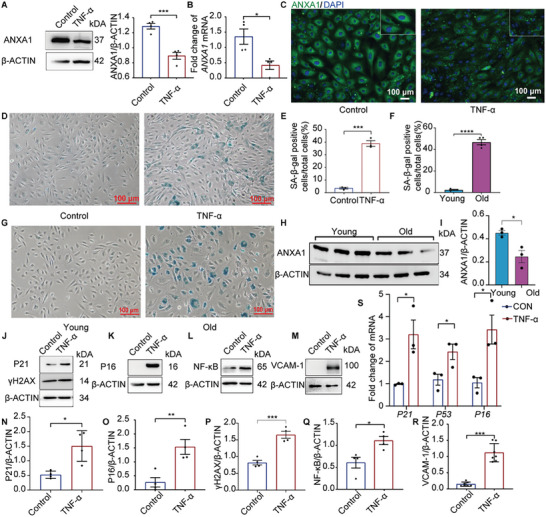
TNF‐α induces senescence and replicative senescence in HUVEC. A) Representative images of ANXA1 and ANXA1 downregulation in the TNF‐α group obtained by semiquantitative western blot analysis (*n* = 4). B) The mRNA expression of ANXA1 was decreased in the TNF‐α group, as semiquantitatively determined by qRT‐PCR (*n* = 4). C) Representative immunofluorescence images showing a decline in ANXA1 expression in the TNF‐α‐induced endothelial senescence group. Bars = 100 µm. D,E) Representative images of SA‐β‐gal staining. The higher number of positive‐stained (blue) cells indicated that cellular senescence was increased in the TNF‐α group (*n* = 3). Bars = 100 µm. F,G) Representative images of SA‐β‐gal staining in HUVECs at different passages. Young HUVECs, passage 7; old HUVECs, passage 25. Positive cells were stained blue and quantified (*n* = 4). The number of positive cells was obviously increased in the old HUVECs group. Bars = 100 µm. H,I) Representative western blot images and semiquantitative analysis of ANXA1 expression showed a decline in ANXA1 expression in the replicative senescence model (*n* = 3). Young HUVECs and old HUVECs are denoted as described above. J–R) The protein levels of P21, γH2AX, P16, NF‐κB, and VCAM‐1 were elevated in the TNF‐α‐induced endothelial senescence group (*n* = 4‐7). S) The mRNA transcript levels of P21, P53, and P16 (*n* = 3) were elevated in the TNF‐α group. The data are presented as the means ±SEMs. Two‐tailed unpaired Student's *t*‐test (A,B,E,F,I,N,S) was used for the analyses. *p < 0.05, **p < 0.01, ***p < 0.001, ****p < 0.0001. ns, *p* > 0.05.

To estimate ANXA1 levels during vascular aging in vitro, HUVECs were treated with TNF‐α (20 ng mL^−1^) for 7 days. A replicative senescence model was established as a positive control. Protein expression and mRNA levels revealed decreased ANXA1 expression in the TNF‐α group in the vascular aging model (**Figure** **7**A–C) and the replicative senescence model (Figure 7H,I). Moreover, SA‐β‐gal staining showed that long‐term inflammation induced cell senescence, similar to the results in the replicative senescence model (Figure 7D–G). The protein expression of P21 and P16 and the mRNA transcript levels of P21, P53, and P16 were increased after 20 ng mL^−1^ TNF‐α treatment (Figure 7J–R). In addition, double‐stranded DNA damage is a well‐known characteristic of senescence.^[^ ^23^ ^]^ Phosphorylation of histone‐H2AX (γ‐H2AX), a marker of DNA damage, was significantly increased in the TNF‐α group (Figure 7J,P). Furthermore, the levels of SASP factors, such as IL‐6, NF‐κB, inhibitor of NF‐κB, ICAM‐1, vascular cell adhesion molecular‐1 (VCAM‐1), and IL‐1β, were elevated in the TNF‐α group (Figure S9, Supporting Information). These results showed a decrease in ANXA1 expression accompanied by inflammation and natural aging processes.

### 2.7 Downregulation of ANXA1 Promotes Vascular Endothelial Cell Senescence

**Figure 8:**
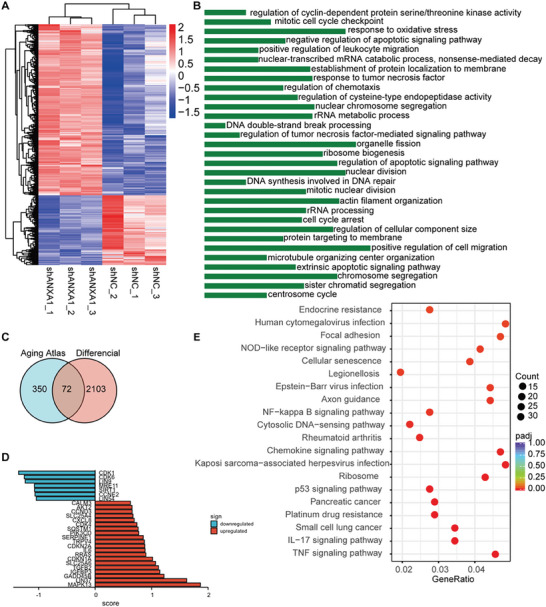
RNA sequencing reveals that knockdown of ANXA1 leads to a senescence phenotype in HUVECs. A) DEG clusters in the sh‐NC and sh‐ANXA1 groups are shown in a heatmap. B) Top 30 enriched GO terms. C) Intersection of genes from the Aging Atlas and DEGs identified by our RNA sequencing analysis. D) Overlapping age‐associated genes are shown with respect to upregulated terms and downregulated terms. E) KEGG enrichment analysis revealed that ANXA1 knockdown was correlated with the cell senescence pathway.

To investigate whether ANXA1 plays an important role in endothelial senescence, lentiviral vectors expressing sh‐ANXA1 and its negative control shRNA (sh‐NC) were applied to reduce ANXA1 expression in HUVECs (Figure S10A,B,E, Supporting Information). RNA sequencing was performed in ANXA1 knockdown HUVECs and control HUVECs. Based on the correlation coefficients (>0.87) between gene expression levels among the samples, the selection of experimental samples was reliable and consistent (Figure S12A, Supporting Information). Three samples of both sh‐NC and sh‐ANXA1 cells were analyzed to identify differentially expressed genes (DEGs). Among the DEGs, 2067 were upregulated, while 2623 were downregulated (Figure S12B, Supporting Information; log2fold change > 0.5, padj < 0.05). Cluster analysis of the DEGs was performed on the sh‐ANXA1 group and the control group (**Figure** **8**A). Gene Ontology (GO) enrichment analysis of the DEGs was performed, revealing several changes in 44 biological processes, 30 cellular components, and 6 molecular function categories (the top 30 are shown in Figure 8D). Kyoto Encyclopedia of Genes and Genomes (KEGG) functional enrichment analysis revealed that ANXA1 was strongly correlated with cellular senescence, the P53 signaling pathway, and several inflammatory pathways, such as the TNF signaling pathway, chemokine signaling pathway, and NF‐κB signaling pathway (Figure 8E). Aging‐related gene sets, including 422 genes from the Aging Atlas,^[^ ^24^ ^]^ were obtained and intersected with the DEGs selected above to identify 72 overlapping genes (Figure 8C). Among these DEGs, the SASP factors Cxcl8, Il‐6, and Tgfβ2 and the senescence‐related markers serine proteinase inhibitor‐1 (Serpine1), cyclin dependent kinase inhibitor (Cdkn)1a, Cdkn2a, and insulin‐like growth factor binding protein 3 were upregulated in the ANXA1‐deficient group (Figure 8D). To verify the sequencing results, we tested both the protein expression and transcript levels of P21 and found that they increased significantly after ANXA1 knockdown (Figure S10A,C,H, Supporting Information). Furthermore, the mRNA expression of SASP factors was elevated in the sh‐ANXA1 group (Figure S,10H, Supporting Information).

To further explore the correlation of ANXA1 expression with aging, SA‐β‐gal staining was performed and the number of positive cells was significantly higher in the knockdown group than in the control group (Figure S10F,G, Supporting Information). The number of cell population doublings showed an obvious decrease in the ANXA1 knockdown group on day 5 (Figure S10D, Supporting Information). Moreover, G0/G1 arrest was observed in the sh‐ANXA1 group (Figure S11A,B, Supporting Information). The migration distance of HUVECs was observably decreased in the sh‐ANXA1 group 48 h after wounding (Figure S11C,D, Supporting Information). Moreover, the tube formation assay showed that ANXA1 knockdown endothelial cells lost the ability to form complete tubes (Figure S11E,F, Supporting Information). These results showed that the downregulation of ANXA1 led to HUVEC senescence.

### 2.8 The ANXA1 Mimetic Peptide Ac2‐26 and Overexpression of ANXA1 in HUVECs Alleviate Age‐Related Alterations

**Figure 9:**
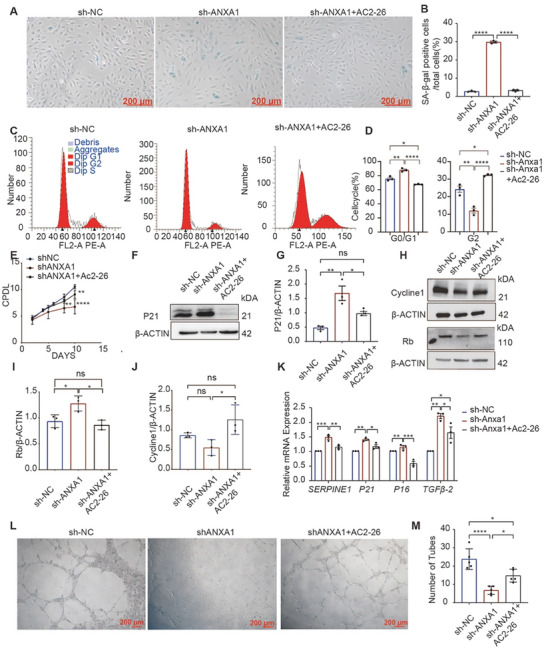
Inflammaging in ANXA1 knockdown HUVECs is reversed by Ac2‐26 treatment. A,B) Representative photographs of SA‐β‐gal staining demonstrating that Ac2‐26 can attenuate senescence in HUVECs (*n* = 3). C,D) G0/G1 arrest was reversed by Ac2‐26 treatment, as determined by flow cytometric analysis (*n* = 3). E) Cumulative population doubling level (CPDL) of the three groups. F,G) Representative western blot images showing that the expression of P21 was reduced by Ac2‐26 treatment (*n* = 3). H–J) Representative western blot images showing that the expression of Rb and Cyclin E1 were reduced by Ac2‐26 treatment (*n* = 3). K) mRNA transcript levels of SERPINE1, P21, P16, and SASP as the TGFβ2 level decreased with Ac2‐26 treatment, as quantified by qRT‐PCR (*n* = 3). L,M) Representative images of the tube formation assay. The number of tubes formed by HUVECs was restored by Ac2‐26 treatment (*n* = 5). The data are presented as the means ±SEMs (B,D,E,G,I,J,K) and means ±SDs (M). One‐way ANOVA followed by the Bonferroni post hoc test (B,D,E,G,I,J,K) was used for the analyses. Bars = 200 µm. *p < 0.05, **p < 0.01, ***p < 0.001, ****p < 0.0001. ns, *p* > 0.05.

To verify the protective function of ANXA1, we treated ANXA1‐deficient HUVECs with 1 µм Ac2‐26 for 3 days and found that Ac2‐26 not only decreased cell senescence (**Figure** **9**A,B) but also rejuvenated cells with G0/G1 arrest in the sh‐ANXA1 group (Figure 9C,D). The number of cell population doublings increased obviously in the shANXA1‐treated Ac2‐26 group (Figure 9E). Both the protein expression and transcript levels of P21, and protein expression of CyclinE1 and Rb decreased significantly with Ac2‐26 treatment compared to those in the sh‐ANXA1 group (Figure 9F–J). Ac2‐26 rescued the senescence process by decreasing the mRNA expression of P21, P16, SERPINE1, and transforming growth factor beta‐2 (TGFβ2) (Figure 9G). Moreover, the migration ability and tube formation function of HUVECs were improved in the Ac2‐26 group compared to the sh‐ANXA1 group (Figure 9L,M,Figure S13A,B, Supporting Information). These results indicated that Ac2‐26 rejuvenated senescent endothelial cells in the ANXA1 knockdown group through the resolution of the SASP.

**Figure 10:**
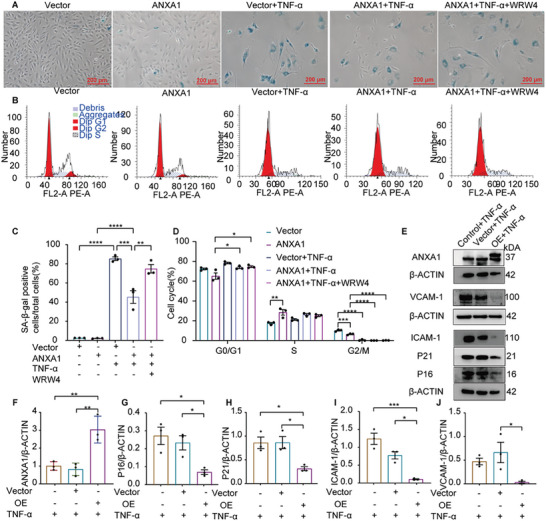
Overexpression of ANXA1 in HUVECs indicates that ANXA1 may exert an anti‐inflammaging effect through its receptor: A,C) Representative photographs of SA‐β‐gal staining and quantification of the positive area indicated that overexpression of ANXA1 in HUVECs reversed TNF‐α‐induced senescence, while treatment with an inhibitor of its receptor FPR2 (WRW4) abolished this beneficial effect (*n* = 3). Bars = 200 µm. B,D) Alleviation of cell cycle arrest was observed in the ANXA1 overexpression group by flow cytometry (*n* = 3). F–J) Representative western blot images showing that the expression levels of P21, P16, VCAM‐1, ICAM‐1, and ANXA1 were significantly decreased in the ANXA1 overexpression treating with TNF‐α group (*n* = 3). The data are presented as the means ±SEMs. One‐way ANOVA followed by the Bonferroni post hoc test (C,D,F–J) was used for the analyses. *p < 0.05, **p < 0.01, ***p < 0.001, ****p < 0.0001. ns, *p* > 0.05.

To fully determine the exact impact of ANXA1 on cell senescence, we also used lentiviral vectors to upregulate ANXA1 (**Figure** **10**E,F) to explore whether an increase in ANXA1 expression can offset the process of cell senescence induced by 7 days of treatment with 20 ng mL^−1^ TNF‐α. We considered that the most likely receptor for ANXA1, formyl peptide receptor‐2 (FPR2),^[^ ^25^ ^]^ might contribute to the important role of ANXA1 in alleviating senescence. WRW4, an antagonist of FPR2, was applied to prohibit ANXA1‐FPR2 communication. Cells with ANXA1 overexpression showed a decline in positive SA‐β‐gal staining during inflammaging, while WRW4 ameliorated this effect (Figure 10A,C). Moreover, flow cytometry showed that ANXA1 upregulation rescued cell cycle arrest in the inflamed environment compared to that in cells without TNF‐α treatment; however, WRW4 did not exacerbate cell cycle arrest (Figure 10B,D). The expression of P21, P16, and adhesion molecules, such as VCAM‐1 and ICAM‐1 on cells, was reduced to some extent with the upregulation of ANXA1 (Figure 10E–J). The wound healing assay also showed that ANXA1 upregulation enhanced cell migration. However, inhibition of FPR2 did not result in a large difference in wound width. (Figure S13C,D, Supporting Information). These results indicated that overexpression of ANXA1 may protect endothelial cells from senescence through FPR2. However, the inhibitor of FPR2 did not prohibit cell migration and cell cycle arrest, which means that other possible mechanisms of cell senescence that involve communication with ANXA1 need further investigation.

## 3 Discussion

Vascular aging plays an important role in physiological and pathological processes contributing to aging organs and systems in humans. Notably, the proinflammatory microenvironment provides opportunities for macrovascular and microvascular pathologies.^[^ ^26^ ^]^ With the structural and functional aspects of detection, we have proposed for the first time several distinct roles that ANXA1 plays in vascular aging, demonstrating a potential causal relationship between ANXA1 protein expression and vascular aging. First, the level of serum ANXA1 was decreased in the serum of both humans and mice and ANXA1 expression was decreased in both naturally senescent and inflammation‐induced senescent endothelial cells. Second, young ANXA1^−/−^ mice show a decline in endothelium‐dependent vasorelaxation prior to vascular structure deterioration. Moreover, ANXA1 deficiency leads to the acceleration of vascular aging not only in terms of age‐related vascular remodeling but also in terms of the recession of vascular function in vivo. Third, ANXA1^△EC^ mice were used to examine the important anti‐aging roles of ANXA1 in the endothelium of the aorta. In addition, downregulation of ANXA1 in HUVECs impaired endothelial cell migration ability and induced G0/G1 arrest and loss of tube formation ability. Fourth, vascular impairment in ANXA1*^−/−^* mice, which showed a loss of vascular homeostasis similar to that in 100‐week‐old aging mice in the aorta, was reversed by treatment with Ac2‐26, an ANXA1 mimetic peptide, suggesting the important role of ANXA1 in vascular geroprotection. Furthermore, as further validation, overexpression of ANXA1 in vitro or exogenous supplementation with Ac2‐26 similarly alleviated endothelial cell senescence and chronic inflammation. Taken together, our findings highlight the important regulation of ANXA1 during vascular aging, thus providing novel ideas for anti‐vascular aging strategies.

Inhibition of inflammation has recently been noted as an effective strategy to delay the progression of aging.^[^ ^27^ ^]^ ANXA1 has been investigated in several age‐associated diseases, such as atherosclerosis, diabetes, and neurodegenerative diseases, in which it exerts its anti‐inflammatory effects by inhibiting platelet‐leukocyte aggregation,^[^ ^28^ ^]^ promoting neutrophil clearance,^[^ ^29^ ^]^ and regulating macrophage differentiation.^[^ ^30^ ^]^ However, the role of ANXA1 in aging remains unclear. Studies have determined a relationship between ANXA1 and vascular degeneration in which ANXA1 can suppress inflammation by inhibiting dihydronicotinamide adenine dinuclectide phosphate oxidases in endothelial cells.^[^ ^31^ ^]^ In addition, ANXA1 expression is decreased in HUVECs with acute‐induced inflammation.^[^ ^32^ ^]^ Of importance for microvascular diseases, ANXA1 expression is also decreased in cerebral blood vessels during hypoxia‐ischemia,^[^ ^18^, ^33^ ^]^ suggesting that ANXA1 might be linked to vascular disorders and, if possible, could be able to postpone vascular aging.

In the present study, we measured ANXA1 levels in both human and mouse serum along with ANXA1 expression in both the aortas and found that these levels decreased with increasing chronological age. These findings suggested that ANXA1, which is secreted from immune cells and expressed in innate immune cells,^[^ ^34^ ^]^ could cause a state of hyposecretion with immunosenescence. According to several studies, as the immune system ages, progressive degradation of the immune response efficiency and impairment of the immune microenvironment are observed.^[^ ^35^ ^]^ Moreover, Elena Y et al. found that ANXA1 serum levels were lower in patients with stroke,^[^ ^28^ ^]^ which is also a complication of CVDs, than in healthy controls. This pattern may reflect the identity of ANXA1 as an anti‐inflammatory protein, and its reduction during aging may indicate senescence and a decreased anti‐inflammatory capacity of the whole system. Alternatively, because ANXA1 is closely related to microcalcification of the cardiovascular system and is always contained within extracellular vesicles as a regulator,^[^ ^36^ ^]^ it could be secreted in vesicles around foci of vascular calcification during aging, thus resulting in a reduction in its level in the circulation; however, further verification of this possibility is needed. Similarly, we then used TNF‐α, which is always associated with large amounts of SASP factors that result in a decrease in endothelial function, which triggers an inflammatory loop and contributes greatly to arterial recession, to induce senescence in HUVECs.^[^ ^4^ ^]^ ANXA1 expression was consistently reduced in both the replicative senescence model and under chronic TNF‐α treatment to simulate the inflammaging state in vitro. However, in recent years, except for HUVECs, more kinds of endothelial cells need to be performed since several kinds of endothelial cells, like cultured endothelial cells from rat aorta, human aorta,^[^ ^37^ ^]^ and bovine pulmonary artery,^[^ ^38^ ^]^ can simulate endothelial cells status. In recent years, various studies focused on vascular calcification and vascular aging used these cells for investigation. More types of endothelial cells should be investigated for intact vascular endothelium aging.

Given that central arterial aging is a major contributor to vascular aging, we then investigated the role of ANXA1 in the aortas of ANXA1‐deficient mice in vivo and in sh‐ANXA1‐transduced HUVECs in vitro. Impaired vascular tone, as an obvious decrease in endothelium‐dependent vasodilation, was observed in ANXA1^−/−^ mice at age 68 weeks. However, 12‐week‐old ANXA1*^−/−^* mice showed only dysfunction of aortic endothelium‐dependent relaxation, without any alterations in aortic morphology and structure or arterial stiffness. Our results showed an increase in both PWV and PP, as well as indicators of age‐related arterial remodeling, including enlargement of the vascular lumen, thickening of vessel walls, and accumulation of collagen, which indicated severe vascular remodeling in 68‐week‐old mice with ANXA1 deficiency. These findings were consistent with the changes observed during vascular aging in the aorta. In addition, the levels of inflammatory and aging indicators were elevated in both ANXA1^−/−^ mice and ANXA1 knockdown HUVECs, suggesting that the proresolution ability of the system was decreased. Lack of ANXA1 can cause neutrophil infiltration due to failure to induce neutrophil apoptosis and resolution;^[^ ^39^ ^]^ thus, the recruitment of various chemokines and cytokines cannot be inhibited, which leads to thrombosis^[^ ^13^, ^28^ ^]^ and calcification in vessel walls and can destroy the normal vessel structure, finally resulting in vascular aging accompanied by chronic inflammation. In addition, ANXA1 knockdown influenced cell migration and the normal cell cycle, leading to irreversible cell senescence. Transcriptome analysis revealed that ANXA1 was closely related to cell senescence, the SASP, and proliferation arrest. It was reported^[^ ^40^ ^]^ that downregulation of ANXA1 inhibited cell migration and proliferation in studies on melanoma progression, consistent with our findings regarding cell senescence. Moreover, we found that the lifespan of ANXA1^−/−^ mice decreased to 17 months, prompting our interest in microvascular destruction and recession of the aorta in the context of ANXA1 deficiency in the endothelium.

It has been reported that during the implantation phase, ANXA1^−/−^ female mice showed an exacerbation of inflammation in uterine fluid and increased plasma progesterone. Moreover, as a result of KO crossings, mice lacking AnxA1 have a larger litter size and skew toward females.^[^ ^41^ ^]^ For the elimination of sex differences, we also observed vascular remodeling and dysfunction in female ANXA1^−/−^ mice. However, ANXA1 deficiency promoted the vascular aging process regardless of sex. Further examination of sex hormones should be performed when ANXA1^−/−^ female mice grow old.

Recent studies have revealed that ANXA1 may be related to capillary formation in infant hemangiomas,^[^ ^42^ ^]^ suggesting that ANXA1 plays an important role in maintaining a normal vessel density. Our HUVEC tube formation assay showed that ANXA1 downregulation compromised the tube formation ability of normal endothelial cells, which corresponded to the microvascular rarefaction observed in ANXA1‐depleted mice. Moreover, we observed enlargement of white adipocytes area in epididymal fat; and BAT whitening. All of these features are similar to those observed in aged mice.^[^ ^22^ ^]^ Collectively, this evidence showed that ANXA1 can promote aging in the adipose tissue. The abovementioned phenomenon suggests that the absence of ANXA1 leads to microvascular rarefaction, resulting in chronic inflammatory infiltration—which is defined as inflammaging—in other solid organs. However, the concrete mechanisms involved need to be deeply elucidated.

To further investigate the effect of exogenous supplementation of ANXA1, ANXA1 KO mice were treated with Ac2‐26.^[^ ^43^, ^44^ ^]^ As expected, the degradation of aortic function and structure was reversed due to the anti‐inflammatory effect of Ac2‐26. The levels of inflammation‐ and senescence‐associated indicators were decreased due to the effectiveness of the ANXA1 mimetic, indicating that ANXA1 possesses geroprotective ability. Moreover, treatment with Ac2‐26 abrogated the severe cell senescence, inflammatory infiltration, and loss of migration caused by the downregulation of ANXA1.

Overexpression of ANXA1 in HUVECs further confirmed that ANXA1 exerts an endothelial‐protective effect. However, we found that ANXA1 did not affect cell senescence only through its receptor FPR2, since ANXA1 can bind to more than one receptor, as previously reported.^[^ ^45^ ^]^ There were also studies that found that FPR activation and Ac2‐26 stimulation could induce phagocytes produced ROS.^[^ ^46^ ^]^ ANXA1 worked with FPR2 through the ERK1/2 pathway and protein kinase B to activate neutrophils.^[^ ^47^ ^]^ ANXA1 also binds to FPR2 to activate adenosine monophosphate‐activated protein kinase, therefore inhibiting the downstream mammalian target of rapamycin.^[^ ^48^ ^]^ In our study, we found that ANXA1 affected CyclinE1, P21, and Rb pathways. These pathways above were linked to inflammatory processes, ROS accumulation, autophagy, and so on, which were all closely associated with aging.^[^ ^49^ ^]^ Thus, further studies should be focused on the intrinsic molecular mechanisms.

In conclusion, we suggest that ANXA1 exerts protective effects of resolving inflammation and maintaining normal vascular homeostasis during the aging process, consequently prolonging a healthy lifespan and maintaining intact systems, which represents a novel aspect of geroprotection. Our studies reinforce the idea that vascular aging constitutes the onset of system aging, indicating that supplementation with ANXA1 is a potential approach for preventing vascular aging and opening further avenues for investigation.

## 4 Experimental Section

### Human Serum Samples

Participants were randomly recruited from the physical examination center at Fujian Medical University Union Hospital from June 2019 to December 2020. Healthy donors were grouped by age: young (18 years to 44 years), middle‐aged (45 years to 64 years), and elderly (more than 65 years). The exclusion criteria were as follows: the presence of CVDs, acute infection within 2 weeks, failure to sign an informed consent, etc. All donors signed the informed consent form, and the study was approved by the Ethics Committee of Fujian Medical University Union Hospital (2017KY092).

### Animal Studies

ANXA1 deficient mice (ANXA1^−/−^) on the C57BL/6J background were generated by GemPharmatech and maintained by mating ANXA1^+/−^ males with ANXA1^+/−^ females. EC‐restricted Anxa1 knockout mice ANXA1‐Cre+/–ANXA1 ^loxp/loxp^ (ANXA1^△EC^) on the C57BL/6JGpt background and their littermates ANXA1‐Cre‐/‐ANXA1^loxp/loxp^(Anxa1 ^fl/fl^) mice were used in the experiments (GemPharmatech Co Ltd).  Flox genotyping primers (forward 5′‐GGCCTTCATGTGCTGTGGAGTTAG‐3′; reverse 5′‐GGATGTTTAAGACGACACGTCAACC ‐3′), and Cre genotyping primers (forward 5′‐GCGGTCTGGCAGTAAAAACTATC‐3′; reverse 5′‐GTGAAACAGCATTGCTGTCACTT‐3′) were used.  Heterozygous ANXA1 KO mice (see Supplemental Methods and Table S2, Supporting Information) were subjected to a strict breeding protocol in a specific pathogen‐free environment. Mice were provided food and water ad libitum in an environment with a controlled temperature and humidity on a 12‐h light/dark cycle. Approval for the animal care and handling protocols were approved by the Committee of Experimental Animal Care of Fujian Medical University (FJMU IACUC 2019‐0106). Laboratory animals were cared for and used according to NIH guidelines.

Male ANXA1 KO mice and the corresponding control mice were used for experiments. The mice were randomly divided into five groups: 1) 12‐week‐old ANXA1 KO mice, 2) 12‐week‐old control mice, 3) 68‐week‐old ANXA1 KO mice, 4) 68‐week‐old control mice, and 5) 100‐week‐old C57BL/6J mice. In addition, 36‐week‐old control mice and 36‐week‐old ANXA1 KO mice were treated with Ac2‐26 (HY‐P1098A, MCE, USA) by intraperitoneal injection (1 mg kg^−1^) or with vehicle (PBS) every other day for 4 months. Female ANXA1 KO mice and the corresponding control mice were also used for experiments. We observed two groups: 1) 68‐week‐old control mice, and 2) 68‐week‐old ANXA1 KO mice.

ANXA1^fl/fl^ and ANXA1^△EC^ mice from 68 weeks old were used for the other part of the experiments.

Mice were weighed and photographed at the end of the experiments after anesthetization by intraperitoneal administration of 30 mg kg^−1^ 1% pentobarbital sodium. Blood was obtained by enucleation, incubated for 30 min at room temperature, and centrifuged at 6000 rpm for 10 min at 4 °C to obtain the serum, which was rapidly stored at −80 °C. Details of the follow‐up procedures are listed in Supplemental Methods, Supporting Information.

### Culture of HUVECs

HUVECs were cultured in a humidified incubator at 37 °C in 5% CO_2_. When the cells were 80–90% confluent, trypsin (S310JV, BasalMedia) was used for serial passaging at a ratio of 1:3. To establish a model simulating inflammaging, HUVECs were treated with 20 ng mL^−1^ TNF‐α (Sigma‐Aldrich, St. Louis, MO, USA) for 7 days to induce the senescence.^[^ ^50^ ^]^ The medium was replaced every other day. Ac2‐26 (MCE, USA) was used at a concentration of 300 µm before lentiviral infection of HUVECs and was applied until 3 days after infection. WRW4 (MCE, USA) was used at 2.5 µm for FPR2 inhibition.

### Statistics

Statistical analyses were performed with GraphPad Prism 9.0. The normal distribution and homogeneity of variance of the data were evaluated using the Shapiro–Wilk normality test and Levene method, respectively. Normally distributed data with equal variance were analyzed with a 2‐tailed Student's *t*‐test for comparisons between two groups, while 1‐way ANOVA followed by the Bonferroni post hoc test was used for comparisons among three or more groups. Data with a nonnormal distribution or unequal variance were analyzed by the Mann–Whitney *U* test (2‐group comparisons) or Kruskal–Wallis test followed by the Nemenyi post hoc test (multiple‐group comparisons).

*N* represents the number of biological replicates. *p* < 0.05 was considered to indicate a significant difference.

The data underlying this article are available in the article and its online supplementary material.

## Conflict of Interest

The authors declare no conflict of interest.

## Author Contributions

Q.Y., Y.K., and X.C. contributed equally to this work. H.H. conceived, designed, and directed this project. Y.K., Q.Y., and X.C. designed and conducted the majority of the experiments. D.L., L.C., and J.Y. assisted with experiments on human serum samples. W.R. assisted with cloning experiments. W.Y. assisted with animal experiments. Q.Y. and Y.K. wrote the manuscript.

## Supporting information

Supporting Information

## Data Availability

The data that support the findings of this study are available from the corresponding author upon reasonable request.
