# Supplementary material for: Loss of Endothelial Annexin A1 Aggravates Inflammation‐Induched Vascular Aging
Source: Adv Sci (Weinh). 2024 Feb 15;11(15):2307040. doi: 10.1002/advs.202307040 (PMC11022713; doi:10.1002/advs.202307040)
Supplement: Supplementary file 1 — Supporting Information [file ADVS-11-2307040-s001.pdf]

ADVANCED SCIENCE
Open Access

Supporting Information

for *Adv. Sci.*, DOI 10.1002/advs.202307040

Loss of Endothelial Annexin A1 Aggravates Inflammation-Induced Vascular Aging

*Qinyi You, Yilang Ke, Xiaofeng Chen, Wanhong Yan, Dang Li, Lu Chen, Run Wang, Jie Yu and Huashan Hong**

SUPPLEMENTAL MATERIAL

Loss of Endothelial Annexin A1 Aggravates Inflammation-Induced Vascular Aging

Authors:

Qinyi You[1]†, PhD; Yilang Ke[1]†, PhD; Xiaofeng Chen[1]†, MD; Wanhong Yan[1], MD; Dang Li[1], PhD; Lu Chen[1], MD; Run Wang[1], MD; Jie Yu[1], MD; Huashan Hong[1]*, MD, PhD

Authorship notes: †These authors contributed equally to this work. Email address: 443065214@qq.com; 928762823@qq.com; 1986xiaofeng0012@163.com.

Affiliations:

[1] Department of Geriatrics, Fujian Medical University Union Hospital, Fujian Key Laboratory of Vascular Aging (Fujian Medical University), Fujian Institute of Geriatrics, Department of Cardiology, Fujian Heart Disease Center, Fujian Clinical Research Center for Vascular and Brain Aging, Fuzhou, Fujian, China.

***Corresponding Authors:**

Huashan Hong, MD, PhD, Department of Geriatrics, Fujian Institute of Geriatrics, Fujian Key Laboratory of Vascular Aging (Fujian Medical University), Fujian Medical University Union Hospital, 29 Xinquan Road, Fuzhou, Fujian 350001, China.

Phone: +86-15959159898 Email address: 15959159898@163.com

Authorship notes: †These authors contributed equally to this work. Email address: 443065214@qq.com; 928762823@qq.com; 1986xiaofeng0012@163.com.

I. Supplemental Figure

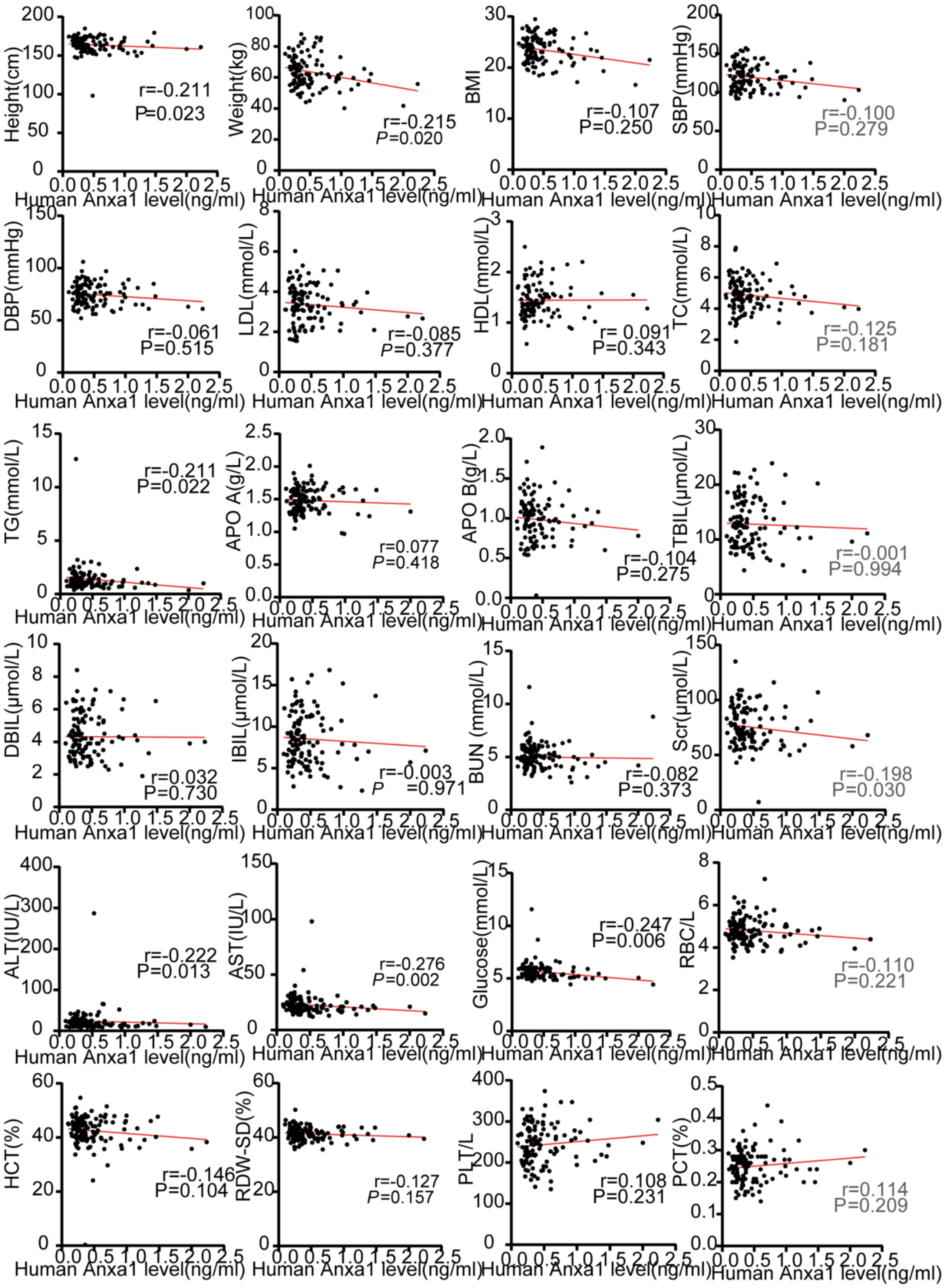

Figure S1. Correlation of ANXA1 expression in serum from healthy humans with clinical characteristics and biochemical and hematological parameters:

(**A**) Correlation of ANXA1 expression with height; r=-0.211, P=0.023. (**B**) Correlation of ANXA1 expression with weight; r=-0.215, P =0.02. (**C**) Correlation of ANXA1 expression with BMI; r=-0.107, P =0.25. (**D-E**) Correlation of ANXA1 expression with SBP and DBP. (**F-S**) Correlation of ANXA1 expression with biochemical parameters. (**T-X**) Correlation of ANXA1 expression with hematological parameters. Spearman rank correlation analysis was used for the analyses. $0<|r|<0.2$ indicates a very weak correlation, $0.2<|r|<0.4$ indicates a weak correlation, and $0.4<|r|<0.6$ indicates a moderate correlation.

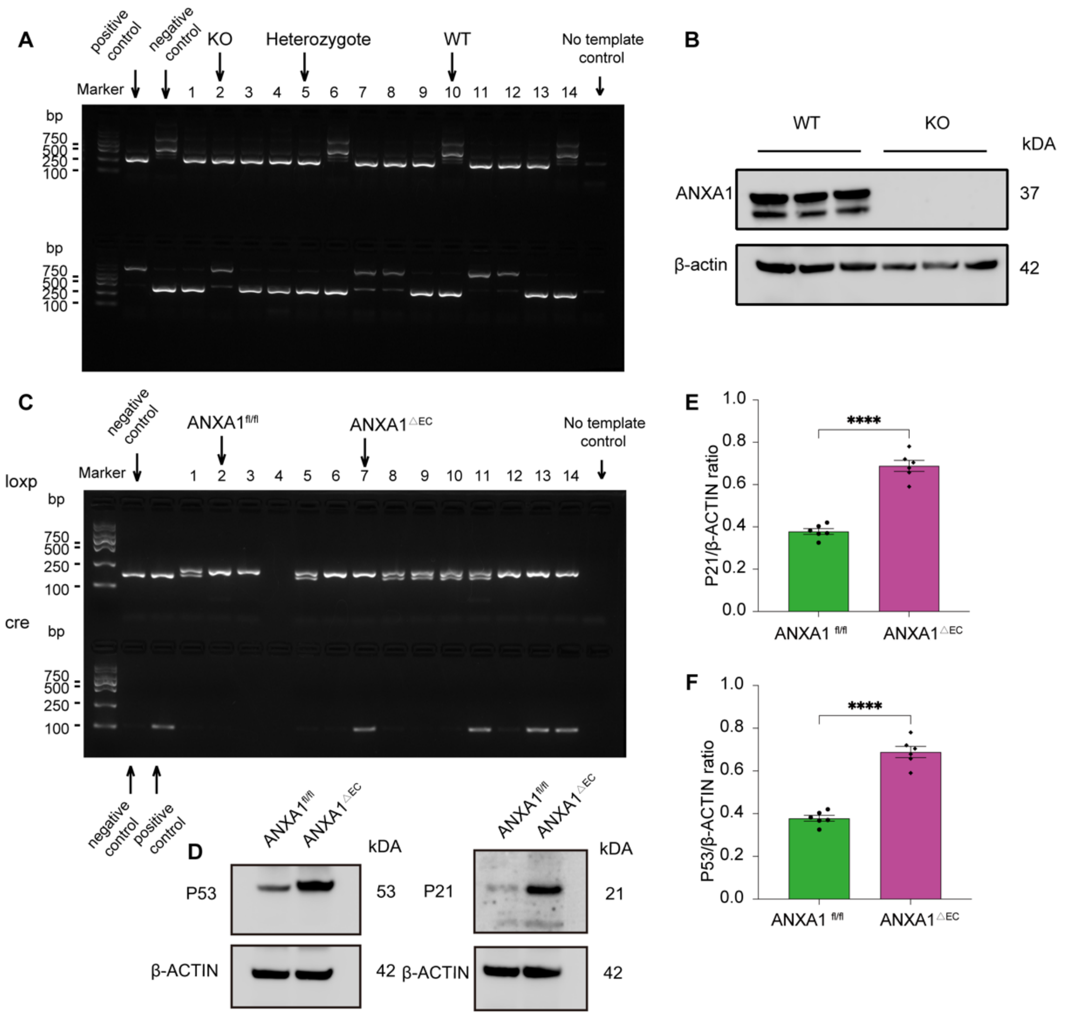

Figure S2. Verification of ANXA1 KO and ANXA1$^{\Delta EC}$ mice:

(**A**) Representative image of DNA extracted from ANXA1 KO mice, run on an agarose gel and stained with nucleic acid stain. DNA from WT mice showed a single WT band at 419 bp, while DNA from KO mice showed a single KO band at 299 bp. Negative, positive and no-template controls were used as references. (**B**) Representative western blot images and semiquantitative analysis of ANXA1 expression (n=3). The knockout efficiency was confirmed by western blotting. (**C**) Representative image of DNA extracted from mice derived from littermates, run on an agarose gel and stained with nucleic acid stain. DNA from ANXA1$^{\triangle EC}$ mice showed a band at 195 bp and 100bp

separately, while DNA from ANXA1$^{fl/fl}$ mice showed a single band at 195 bp. Heterozygote mice showed double bands. Negative, positive and no-template controls were used as references. (**D-F**) Representative western blot images showing that the expression levels of P53 and P21 were significantly increased in the aortas of ANXA1 $^{\triangle EC}$ mice (n=6). The data are presented as the means ±SEMs. The two-tailed unpaired Student's *t* test was used for the analyses. ****P<0.0001.

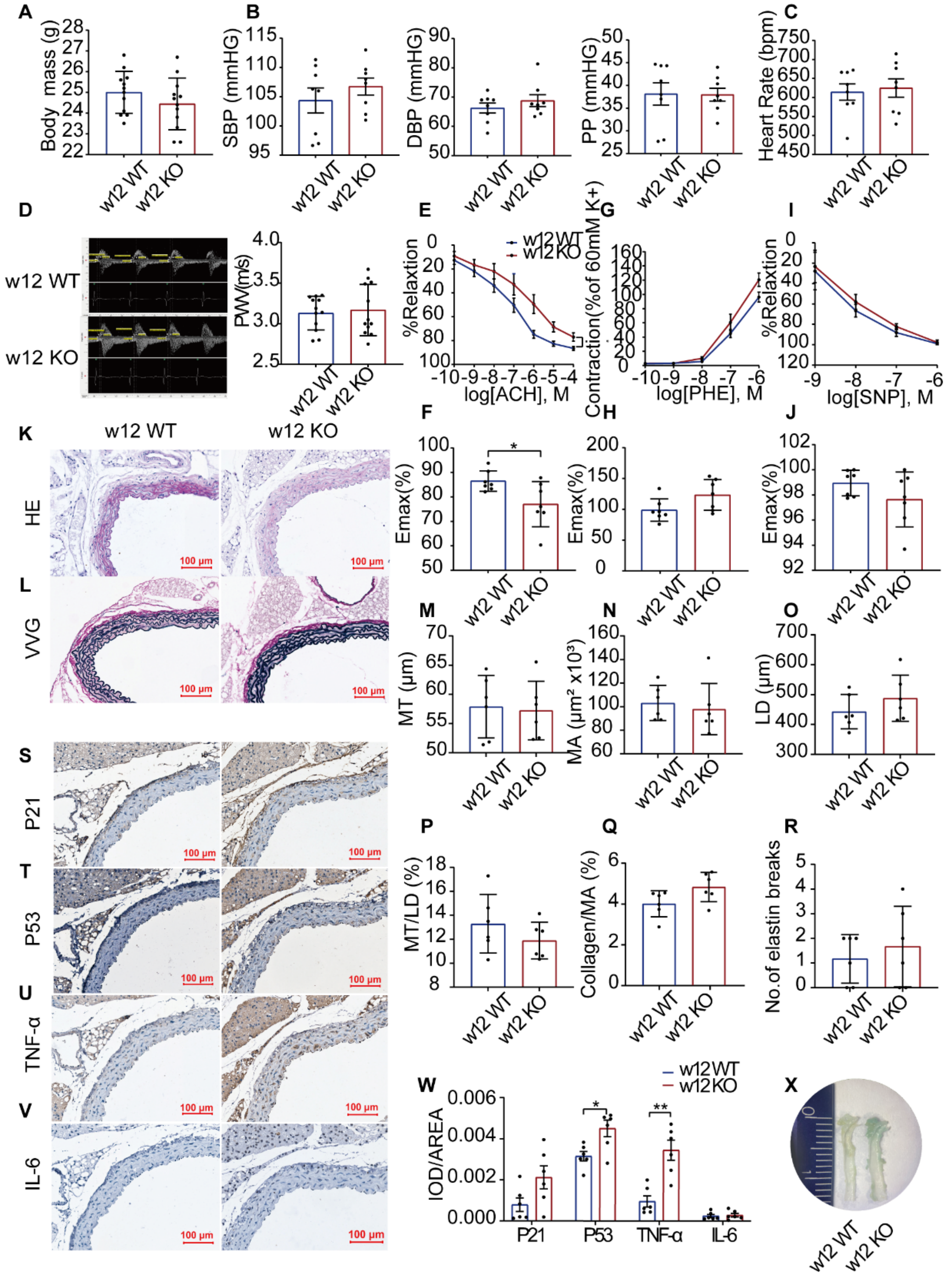

Figure S3. Evaluation of vascular aging in 12-week-old ANXA1 KO mice:

(**A**) Body mass of WT and KO mice at 12 weeks of age (n=12). (**B**) SBP, BP and PP of WT and KO mice (n=8) at 12 weeks of age. (**C**) HR of WT and KO mice (n=8).

(**D**) A noninvasive Doppler blood flow monitor was used for PWV measurement (n=12). (**E**-**F**) Endothelium-dependent relaxation of thoracic aortic rings was reduced in KO mice (12 weeks). Relaxation was induced by ACH (n=6). (**G-H**) Contraction of thoracic aortic rings was induced by PHE (n=6). (**I-J**) Endothelium-independent relaxation of thoracic rings was induced by SNP (n=6). (**K-R)** Vascular remodeling indicators, such as the MT, MA, LD, MT/LD, collagen area/MA and number of elastin breaks, were analyzed by HE staining and VVG staining of mouse aortas. Bars=100 μm. (**S-W**) Representative images of staining for senescence indicators, such as P21 and P53, and senescence-associated inflammatory cytokines, such as TNF-α and IL-6, in aortas from ANXA1 KO mice and control mice (12 weeks). Expression was detected by immunohistochemical staining (n=6). Bars=100 μm. ANXA1 KO mice showed higher expression of P53 and TNF-α. (**X**) Representative images of SA-β-gal staining in the aortas of WT and KO mice (n=6). The data are presented as the means ±SEMs (**B-D, W**) and means ±SDs (**A, E-J** and **M-R**). Two-tailed unpaired Student's *t* test (**A-B, D, F, H, J, M-Q** and **W**), the Mann–Whitney U test (**C, R**), and multiple repeated measures ANOVA followed by the Bonferroni post hoc test (**E, G** and **I**) were used for the analyses. $*P<0.05$, $**P<0.01$, $***P<0.001$, $****P<0.0001$; $P>0.05$ is not indicated.

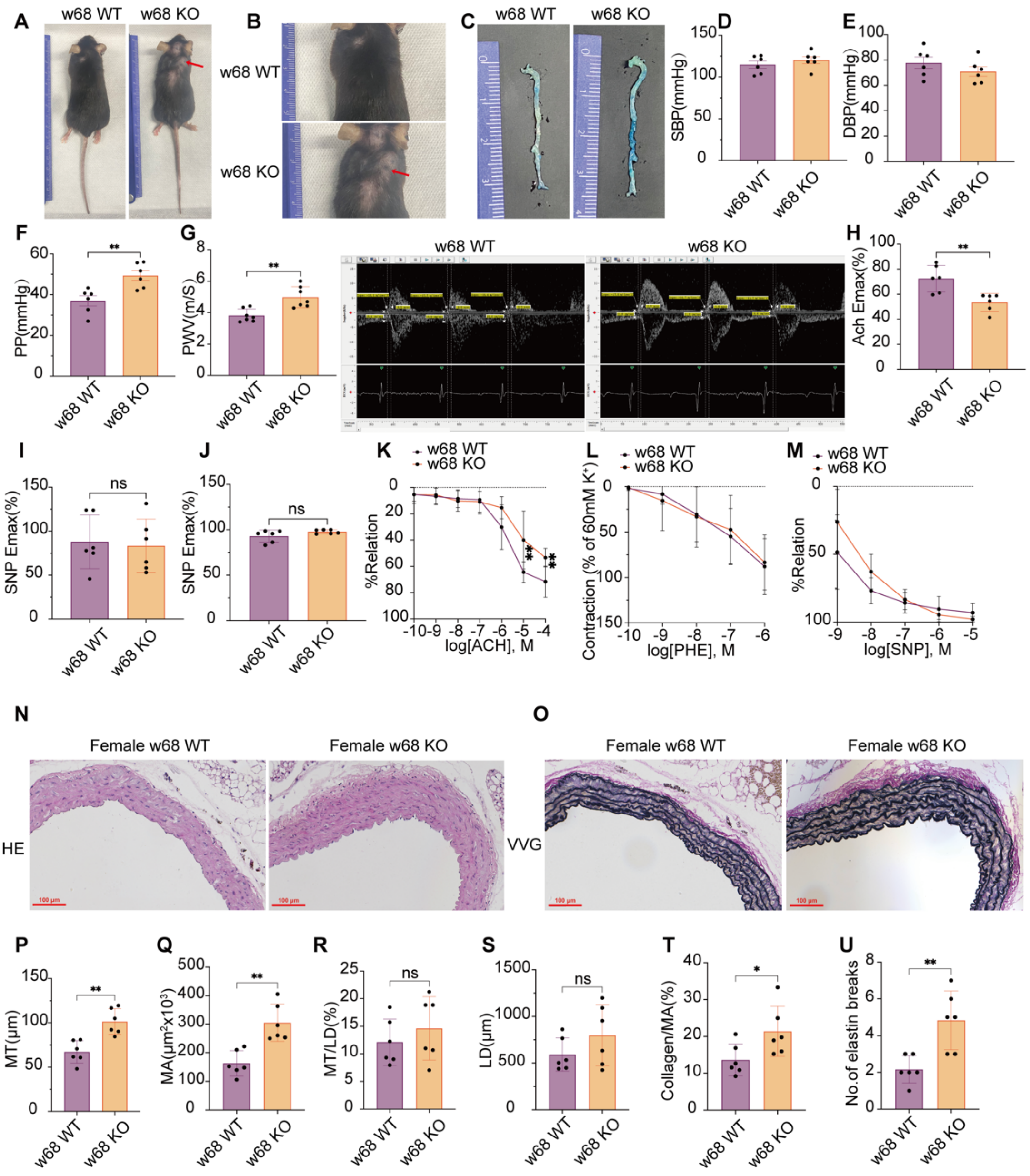

Figure S4. Evaluation of vascular aging in 68-week-old ANXA1 KO female mice: (**A-B**) The fur and hair of female WT and KO mice at 68 weeks of age (n=6). (**C**) The area of positive SA-β-gal staining in the aorta was increased in female ANXA1 KO mice at 68 weeks of age (n=6). (**D-F**) SBP, BP and PP of WT and KO female mice at 68 weeks of age (n=6). (**G**) The PWV increased significantly in female KO mice at 68 weeks of age. (n=6). (**H** and **K**) ACH-induced endothelium-dependent relaxation of

thoracic aortic rings in female KO mice at 68 weeks of age. Maximum relaxation induced by ACH (n=6). (**I and L**) Contraction of thoracic aortic rings was induced by PHE (n=6). (**J and M**) Endothelium-independent relaxation of thoracic rings was induced by SNP (n=6). (**N-U**) Vascular remodeling indicators, such as the MT, MA, LD, MT/LD, collagen area/MA and number of elastin breaks, were analyzed by HE staining and VVG staining of mouse aortas. Bars=100 μm. The data are presented as the means ±SEMs (**D-G**) and means ±SDs (**H-M** and **P-U**). Two-tailed unpaired Student's *t* test (**D-J** and **P-U**) and multiple repeated measures ANOVA followed by the Bonferroni post hoc test (**K-M**) were used for the analyses. Bars=100 μm. **P*<0.05, ***P*<0.01, ****P*<0.001, *****P*<0.0001. ns, *P*>0.05.

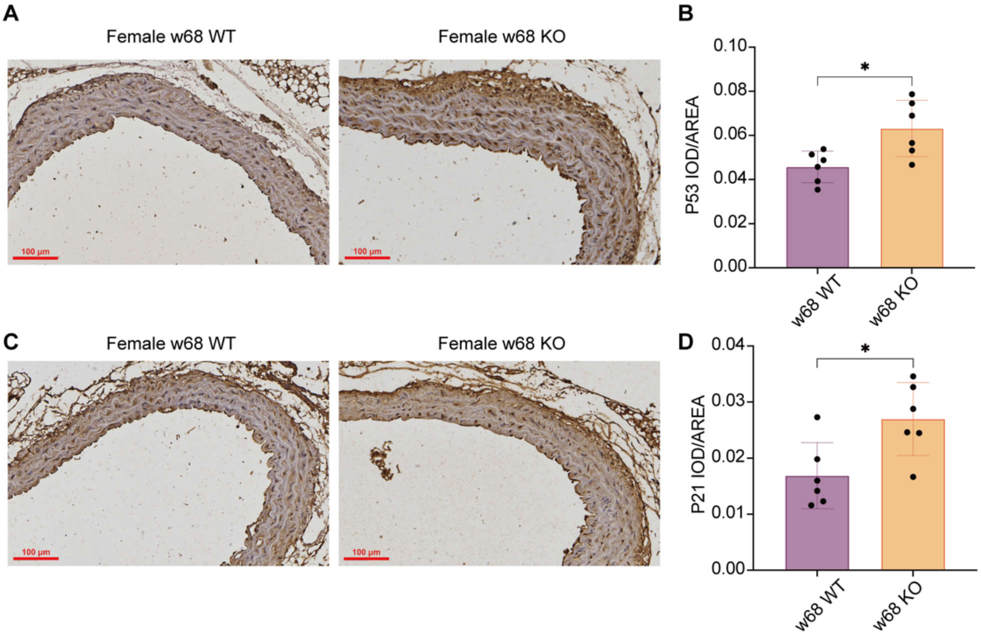

Figure S5. Senescence protein expression of aorta in 68-week-old ANXA1 KO female mice:

(**A-B**) Representative images of immunochemistry staining for P53 in 68-week-old ANXA1 KO female mice. (**C-D**) Representative images of immunochemistry staining for P21 in 68-week-old ANXA1 KO female mice. Bars= 100 μm. The data are presented as the means ±SDs (**A-D**). Two-tailed unpaired Student's *t* test (**B** and **D**) was used for the analyses. **P*<0.05. ns, *P*>0.05.

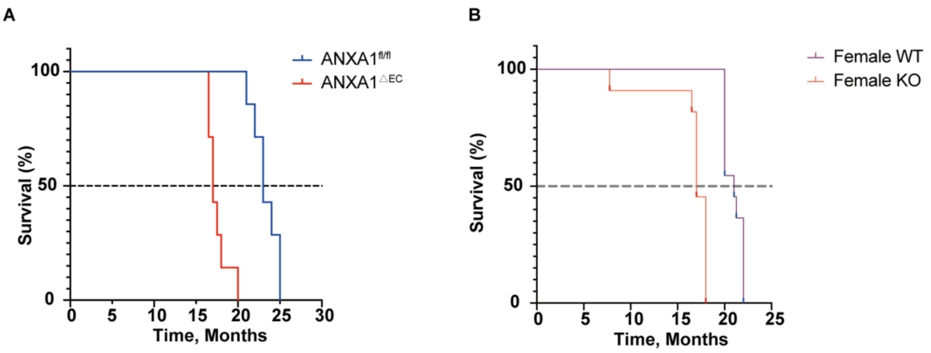

Figure S6. Survival rate of ANXA1$^{\triangle EC}$ and female ANXA1 knockout mice.

(**A**) Survival rate of ANXA1$^{fl/fl}$ and ANXA1$^{\triangle EC}$ mice. (**B**) Survival rate of female ANXA1 KO mice. The data are presented as the means ± SDs (A-B). Kaplan–Meier survival analysis (A-B) was used for the analyses.

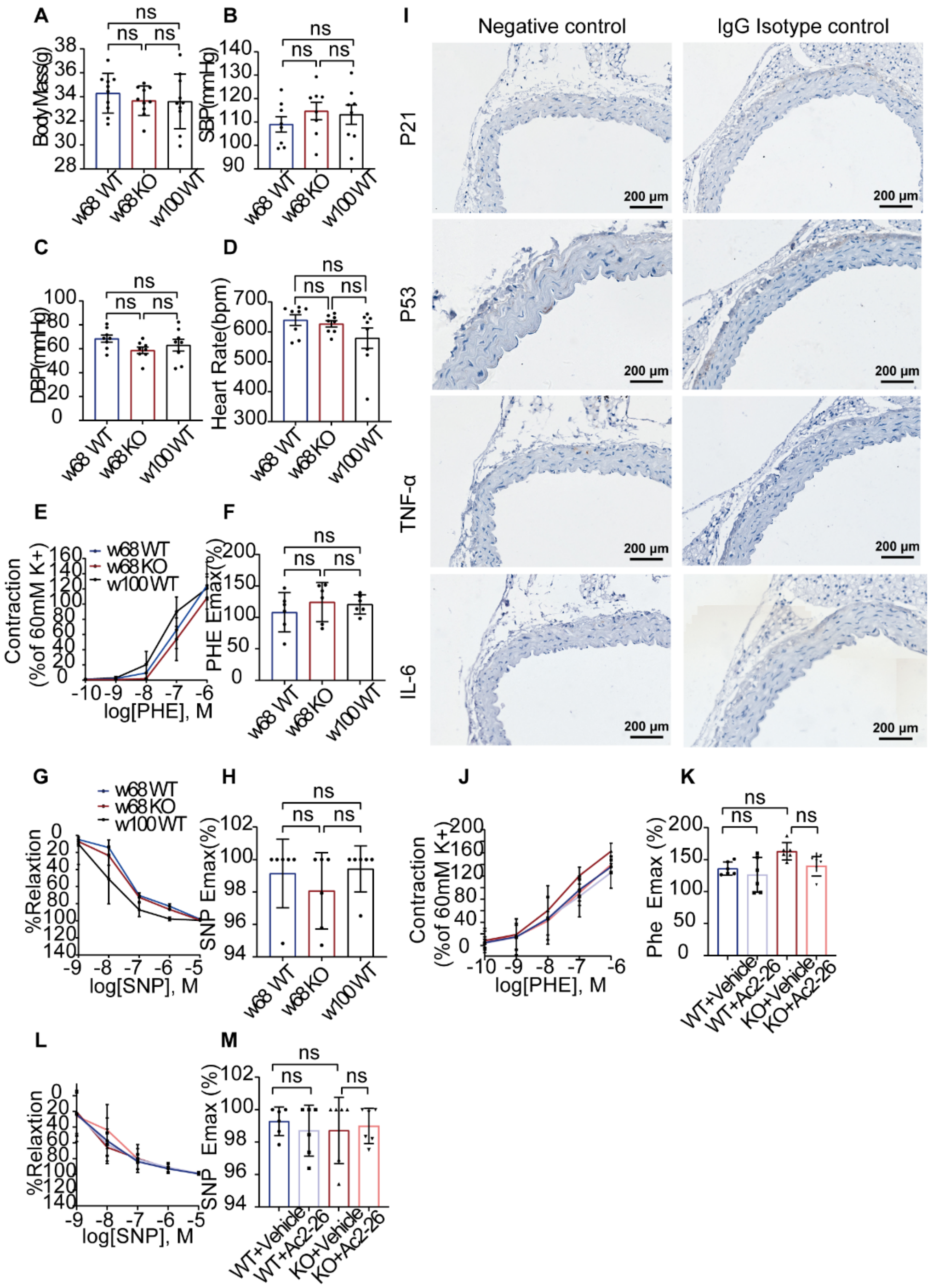
A
ns
ns ns
BodyMass(g)
w68 WT
w68 KO
w100 WT
B
SBP(mmHg)
C
DBP(mmHg)
D
Heart Rate(bpm)
E
Contraction (%of 60mM K+)
log[PHE], M
F
PHE Emax(%)
G
%Relaxtion
log[SNP], M
H
SNP Emax(%)
I
Negative control
IgG Isotype control
P21
P53
TNF-α
IL-6
200 μm
J
Contraction (%of 60mM K+)
log[PHE], M
K
Phe Emax (%)
WT+Vehicle
WT+Ac2-26
KO+Vehicle
KO+Ac2-26
L
%Relaxtion
log[SNP], M
M
SNP Emax (%)

Figure S7. ANXA1 deficiency did not alter body mass, blood pressure, HR, aortic contraction or endothelium-independent aortic relaxation:

(**A**) Body mass of WT and KO mice (68 weeks) and old mice (100 weeks) (n=10). (**B-D**) SBP, DBP and HR in w68 WT mice, w68 KO mice and w100 old mice (n=8). (**E-F**) Contraction of thoracic aortic rings was induced by PHE (n=6). The maximum contraction of thoracic aortic rings did not differ significantly among the indicated 3 groups. (n=6). (**G-H**) Endothelium-independent relaxation of thoracic rings was induced by SNP (n=6). The maximum endothelium-independent relaxation of thoracic rings did not differ significantly among the 3 groups. (n=6). (**I**)Representative images of the negative control and IgG isotype control for P21, P53, TNF-α, and IL-6. Bars= 200 μm. (**J-K**) PHE induced contraction of thoracic aortic rings (n=6). (**L-M**) Endothelium-independent relaxation of thoracic rings was induced by SNP (n=6). The data are presented as the means ±SEMs (**B, C** and **D**) and means ±SDs (**A, E-H** and **J-M**). One-way ANOVA followed by the Bonferroni post hoc test (**A-C, F** and **H**), two-way ANOVA followed by the Bonferroni post hoc test (**K** and **M**), the Kruskal–Wallis test (**D**) and multiple repeated measures ANOVA followed by the Bonferroni post hoc test (**E, G, J** and **L**) were used for the analyses. *$P<0.05$, **$P<0.01$, ***$P<0.001$, ****$P<0.0001$.

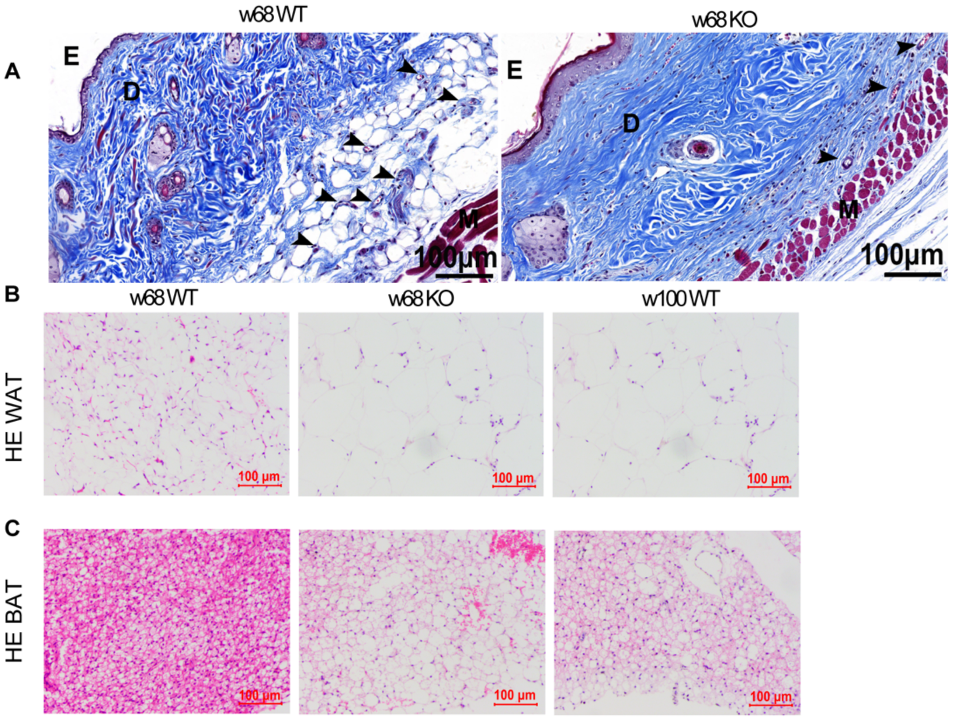

Figure S8. ANXA1 deficiency alterations among subdermal microvascular density, white adipose tissue and brown adipose tissue.

(A) Representative images of Masson-Trichrome–stained of skin (arrowheads showed subdermal capillaries) indicated that 68 weeks KO mice had less subdermal microvascular density. E, epidermis, D, dermis, M, muscle. **(B-C)** H&E staining of WAT and BAT tissue in mice from WT and KO (68 weeks) and old mice (100 weeks) (n=6). Bars =100 µm. BAT whitening was observed in KO and old mice.

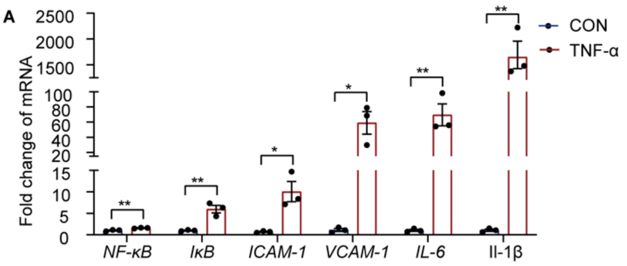

Figure S9. Age-related and inflammatory mRNA was elevated in TNF-α group:

(**A**) The mRNA transcript levels of *NF-κB, IκB, ICAM-1, VCAM-1, IL-6 and IL-1β* (n=3) were elevated in the TNF-α group. The data are presented as the means ±SEMs. Two-tailed unpaired Student's *t* test was used for the analyses. *P<0.05, **P<0.01. ns, P>0.05.

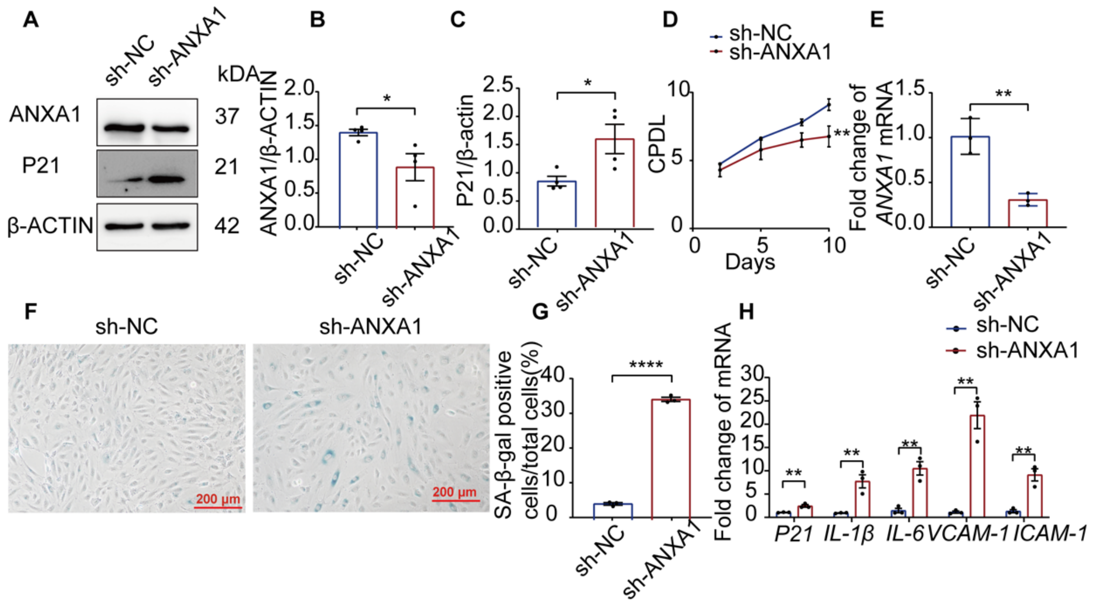

Figure S10. ANXA1 knockdown facilitated the senescence and inhibited the proliferation of HUVECs:

(**A-C**) Representative western blot images of ANXA1 and P21 expression. Semiquantitative analysis in HUVECs indicated downregulation of ANXA1 and increased expression of P21 in the sh-ANXA1 group (n=4). (**D**) The number of cumulative population doublings (CPDL) was decreased in the ANXA1 knockdown group during the first 10 days of culture (n=3). (**E**) Verification of decreased *ANXA1* mRNA expression in the ANXA1 knockdown group by qRT–PCR (n=3). (**F-G**) Increased cell senescence, shown by the increase in the number of cells with positive SA-β-gal staining in the ANXA1 knockdown group, was quantified (n=3). (**H**) Higher mRNA expression levels of *P21, IL-6, IL-1β, VCAM-1* and *ICAM-1* were observed in the sh-ANXA1 group (n=3). The data are presented as the means ±SEMs (**B-E** and **G-H**). Two-tailed unpaired Student's t test (**B-E** and **G-H**) was used for the analyses. Bars=200 μm. *P<0.05, **P<0.01, ***P<0.001, ****P<0.0001. ns, *P*>0.05.

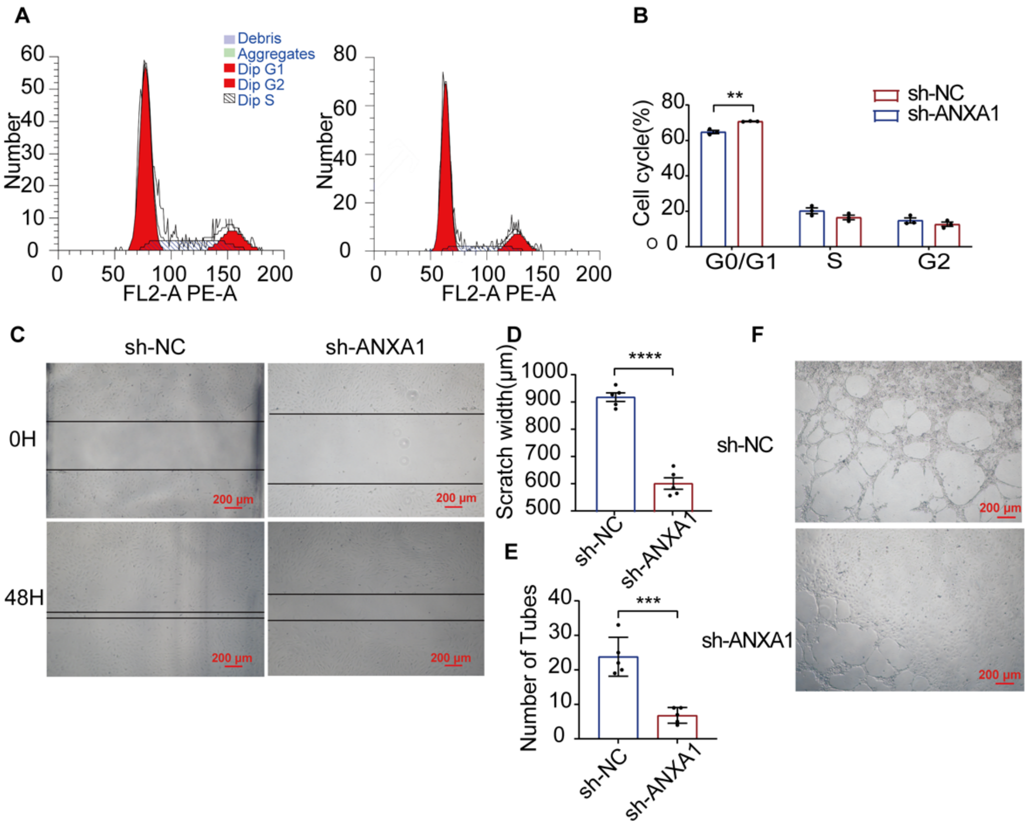

Figure S11. Function compromised was observed in ANXA1 knockdown HUVECs.

(**A-B**) Representative images of flow cytometric analysis of the cell cycle distribution indicating G0/G1 arrest in the sh-ANXA1 group. (**C-D**) Representative photographs of the wound healing assay. A reduced migration distance of HUVECs was observed in the sh-ANXA1 group (n=5). (**E-F**) A tube formation assay was performed with ANXA1 knockdown HUVECs (n=5). Tube formation was decreased in the ANXA1 knockdown group. The data are presented as the means ±SEMs (**B** and **D**) and means ±SDs (**E**). Two-tailed unpaired Student's t test (**B, D** and **E**) was used for the analyses. Bars=200 μm. *P<0.05, **P<0.01, ***P<0.001, ****P<0.0001. ns, *P*>0.05.

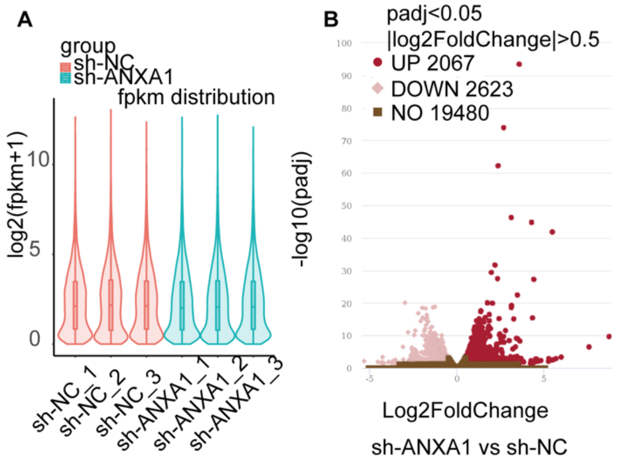

Figure S12. Quality control of ANXA1 knockdown in HUVECs:

(**A**) Quality control of RNA sequencing data is shown. (**B**) Volcano plot showing the 2067 genes upregulated and 2623 genes downregulated in the sh-ANXA1 group.

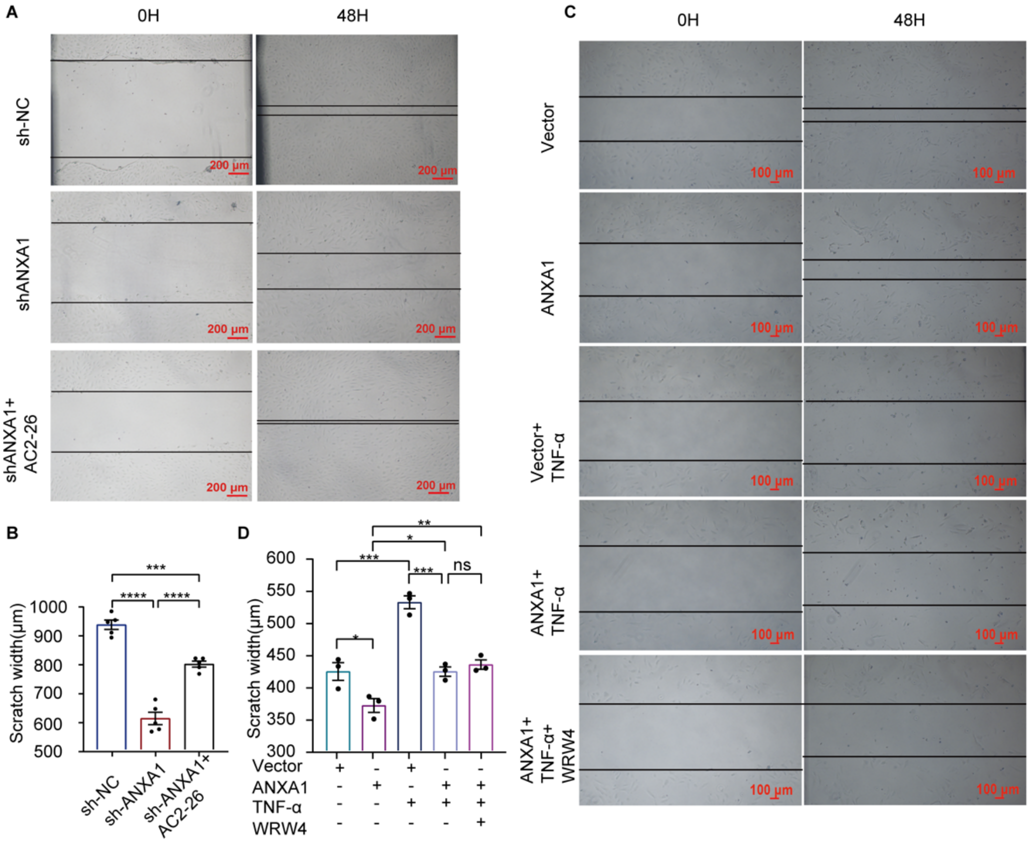

Figure S13. Wound healing assay in ANXA1 knockdown HUVECs reversed by Ac2-26 treatment and overexpression of ANXA1 in HUVECs:

(**A** and **B**) A wound healing assay was performed to assess the migration capacity of HUVECs. After Ac2-26 treatment, the migration ability of HUVECs was restored (n=5). (**C-D**) Representative images of the wound healing assay showed restoration of the migration ability in the ANXA1 overexpression group compared to the TNF-α group. (n=3). Bars=100 µm. The data are presented as the means ±SEMs. One-way ANOVA followed by the Bonferroni post hoc test (**A-D**) was used for the analyses. *P<0.05, **P<0.01, ***P<0.001, ****P<0.0001. ns, *P*>0.05.

II.Supplemental Table

Table S1. Clinical hematological and biochemical parameters in healthy adults

	Young(n=42)	Middle-aged(n=48)	Old(n=43)	*P* value
Age(years)	34.62±0.98	55.77±0.78	34.62±0.98	<0.001
Male (n, %)	18 (42.86)	29 (60.42)	29 (67.44)	0.062
ANXA1 level	0.75[0.61,0.88]	0.38[0.31,0.46]	0.30[0.26,0.33]	<0.001
Height(cm)	163.43±8.49	163.29±12.46	164.31±7.00	>0.05
Weight(kg)	61.22±11.80	64.21±9.07	66.74±9.67	0.032
BMI	22.50±2.99	23.51±2.05	24.66±2.75	0.001
SBP (mmHg)	114.72±12.44	116.79±15.00	129.45±18.64	<0.001
DBP (mmHg)	72.74±9.24	75.52±10.30	75.03±11.51	>0.05
Hypertesion(%)	2 (4.76)	8 (16.67)	13 (30.23)	0.001
Diabetes (%)	2 (4.76)	2 (4.17)	8 (19.05)	0.005
LDL (mmol/L)	3.15±0.79	3.38±0.87	3.51±1.10	>0.05
HDL (mmol/L)	1.55[1.39,1.78]	1.37[1.16,1.59]	1.26[1.2,1.59]	>0.05
TC (mmol/L)	4.62±0.83	4.91±1.00	5.02±1.13	>0.05
TG (mmol/L)	0.86[0.70,1.13]	1.14[0.97,1.89]	1.12[0.97,1.72]	0.001
APO A1 (g/L)	1.51±0.20	1.49±0.20	1.44±0.17	>0.05
APO B (g/L)	0.91[0.75,1.06]	0.98[0.85,1.12]	1.07[0.90,1.20]	0.023
TBIL(μ mol/L)	12.20[9.35,16.63]	12.30[9.05,16.18]	12.30[9.35,14.05]	>0.05
DBIL(μ mol/L)	4.15[9.35,16.63]	4.10[9.35,16.63]	4.10[9.35,16.63]	>0.05
IBIL(μ mol/L)	8.30[9.35,16.63]	8.00[9.35,16.63]	7.70[9.35,16.63]	>0.05
ALT(IU/L) (log-transformed)	1.23±0.32	1.32±0.17	1.30±0.19	>0.05
AST(IU/L) (log-transformed)	1.30±0.15	1.35±0.11	1.35±0.10	>0.05
BUN (mmol/L) (log-transformed)	0.64±0.10	0.70±0.09	0.71±0.10	0.008
Scr(μ mol/L) (log-transformed)	1.85±0.10	1.85±1.18	1.88±0.09	>0.05
Glu(mmol/L) (log-transformed)	0.72±0.04	0.75±0.03	0.77±0.07	0.001
Hb (g/L)	4.74±0.62	4.81±0.44	4.85±0.68	>0.05
HCT (%)	42.40[39.65,45.55]	43.90[39.85,45.50]	43.90[39.85,47.25]	>0.05
PLT (×10^9/L)	259.11±46.47	241.00±48.14	230.69±42.32	0.025
MPV	10.05[39.65,45.55]	10.45[39.65,45.55]	9.80[39.65,45.55]	>0.05
PCT (%)	0.27[0.24,0.29]	0.25[0.21,0.28]	0.22[0.2,0.26]	0.007

Abbreviation: SBP, systolic blood pressure; DBP, diastolic blood pressure; LDL, low density lipoprotein; HDL, high density lipoprotein; TC, total cholesterol; TG, Total triglyceride; APO A1, human apolipoprotein A-1; APO B, human apolipoprotein B; TBIL, total bilirubin; IBIL: indirect bilirubin; DBIL, direct bilirubin; ALT, alanine aminotransferase; AST, aspartate aminotransferase; BUN, Blood Urea Nitrogen; Scr, serum creatinine; Hb, red blood cells; HCT, hematocrit; SD, red blood cell distribution width-standard deviation; CV, red blood cell distribution width-coefficient of variation; PLT, platelet count; MPV, mean platelet volume; PDW, platelet distribution width; PCT, plateletocrit.

Table S2. Animals resource

**Species**	**Vendor or Source**	**Background Strain**	**Sex**	**Persistent ID / URL**
Mouse	Gempharmatech Co., Ltd, Nanjing, China	C57BL/6J	Male	T016864/https://www.gempharmatech.com/

III. Supplemental Methods

Blood pressure measurement

Mice were placed in a heating cylinder, which was covered with a net to confine the mice and keep them warm. Inside the sleeve, blood pressure was measured via the tail with a noninvasive tail sphygmomanometer (Softron, China). Measurements were repeated at least 3 times to ensure stability. The mean systolic blood pressure (SBP), mean diastolic blood pressure (DBP), mean blood pressure and HR were obtained from the machine. The PP was calculated as the difference between the mean SBP and mean DBP.

PWV measurement

Mice were anesthetized by intraperitoneal administration of 1% pentobarbital sodium and fixed on the heating platform along with the electrocardiography (ECG) apparatus. Given the distance, two probes were used simultaneously to acquire Doppler velocity spectrograms for the aortic arch and abdominal aorta separately by a Doppler Flow Velocity System (Indus, USA). The electrocardiographic signal was acquired for measurement of the time interval, and the distance was measured by a ruler. PWV(m/s) =Distance(mm)/Time(ms).

Aortic function assessment

A wire myograph system (DMT620M, ADInstruments, Australia) was used for the assessment of aortic function. The tissue around the vascular rings was removed, and

vessels were mounted onto the wire in a gas-filled chamber (95% O_2 and 5% CO_2) at 37°C. A force of 6 mN was applied to maintain an initial tension recording with LabChart software (version 8). After three equilibration cycles times (20 minutes each), the vessels were subjected to two cycles of contraction by the application of 60 mM high-potassium physiological saline solution. The aortic rings were constricted by the application of PHE (10^{-6} M; Sigma–Aldrich, St. Louis, MO, USA) and relaxed by the application of ascending concentrations of ACH, (10^{-10} –10^{-4} M; Sigma–Aldrich, St. Louis, MO, USA), which simulated endothelial-dependent vasodilation. PHE (10^{-10} to 10^{-6} M) was used to study the constriction of the vascular rings. To evaluate endothelium-independent vasodilation, N_{ω}-nitro-L-arginine methyl ester (L-NAME; 10^{-5} M; Beyotime, Shanghai, China) was applied before induction of constriction by PHE (10^{-6} M), after the cumulative addition of SNP (10^{-9}–10^{-5} M; Sigma–Aldrich, St. Louis, MO, USA).

Details of animal studies

The heterozygotes were purchased from GemPharmatech (Nanjing, China). The offspring of heterozygotes were identified by PCR with nucleic acid gel imaging using DNA extracted from mouse tails with a one-step mouse genotyping kit (Vazyme, China).

Mice were perfused with normal saline through the left ventricle, and the thoracic aorta was immediately harvested and placed in physiological saline solution (PSS) to preserve endothelial activity. The portion of the thoracic aorta located immediately

before the aortic arch was used for the measurement of vascular systolic and diastolic function [20]. The rest of the aorta was fixed with 4% paraformaldehyde overnight. First, slides containing fixed thoracic aorta tissue were sequentially subjected to HE staining, VVG staining, and incubation with antibodies specific for P53, P21, TNF-α and IL-6 for immunohistochemical staining. Abdominal aortas were harvested in liquid nitrogen and stored at -80°C for western blotting. The other 6 mice per group were dissected for harvesting of the thoracic aorta for SA-β-gal staining. Cardiac tissues, with the exception of the aorta, were harvested, and the cardiac apex was stored at -80°C for western blotting, while the rest of the tissues were fixed with 4% paraformaldehyde. The first slide was used for HE staining, and the second slide was used for CD31 immunohistochemical staining. The median lobe of the liver was fixed for paraffin sectioning, the anterior right lobe was fixed for cryosectioning, and the left lateral lobe was frozen at -80°C for western blotting. The left side of epididymal fat, dorsal brown fat, kidney and dorsal skin were obtained for western blotting, while the corresponding right side were obtained for paraffin sectioning.

Enzyme-linked immunosorbent assay (ELISA)

The concentration of ANXA1 in serum from healthy donors was measured by ELISA kits (ab222868, Abcam, UK). The standard and samples were prepared prior to incubation at room temperature and were incubated for 2 hours in wells. The wells were then washed five times, and a biotinylated anti-ANXA1 antibody was added for 1 hour. After washing as described above, the wells were incubated with the streptavidin-

peroxidase conjugate and chromogen substrate for 30 minutes and 15 minutes, respectively. After stop solution was added, the absorbance at 450 nm was measured using a plate reader (Bio-Rad, Hercules, CA, USA). The concentration of ANXA1 in mouse serum was quantified according to the manufacturer's protocols (37) (MBS9407240, MyBioSource, USA). MCP-1 (MJE00B, R&D Systems, Australia) and CRP ELISA kits (MCRP00, R&D Systems, Australia) were used for quantification according to the manufacturer's instructions.

Histopathological examination

Thoracic aortas fixed with 4% paraformaldehyde overnight were deparaffinized with xylene and hydrated in water with decreasing concentrations of ethanol. A HE kit (Servicebio, Wuhan, China) and VVG staining (Servicebio, Wuhan, China) were used to evaluate the vascular wall thickness and elastin breaks along with the collagen area. The MT, MA, MT/LD and percentage of collagen area (collagen/MA) were calculated with IPP7.0 software according to the methods described previously(5).

Immunohistochemical staining

Four-micrometer sections of paraffin-embedded thoracic aortas were deparaffinized with xylene and hydrated with decreasing concentrations of ethanol (100% to 50%). The expression of ANXA1, P53, P21, IL-6, and TNF-α was assessed according to previous protocols (5). A mouse monoclonal anti-IL-6 antibody (1:100, sc-32296), rabbit polyclonal anti-P21 antibody (1:200, GB11153), rabbit polyclonal anti-P53

antibody (1:200, AF0879), rabbit polyclonal anti-TNF-α antibody (1:200, A111534), rabbit monoclonal anti-ANXA1 antibody (1:400, ab214486), mouse IgG isotype control (ab37355) and rabbit IgG isotype control (ab172730) were applied.

Multiplex immunofluorescence staining

A tyramide signal amplification (TSA) kit (Servicebio, Wuhan, China) was applied for multiplex immunohistochemical staining, which was performed to localize and quantify the expression of ANXA1 in the aortas of mice at different ages. Four-micrometer sections of paraffin-embedded thoracic aortas were deparaffinized and hydrated according to the steps as described above. After removal from ethylenediaminetetraacetic acid (pH=9.0), the aortic sections were blocked in 3% bovine serum albumin (BSA) for at least 1 hour at room temperature and incubated with the primary antibody at 4°C overnight. A horseradish peroxidase (HRP)-conjugated secondary antibody was then applied for 50 minutes at room temperature and was replaced by TSA staining solution. A microwave was used for antibody removal. The other primary antibodies were applied according to the manufacturer's instructions.

Masson Trichrome Assay

4μm sections of paraffin-embedded skin sections were dewaxed to water as previous described. Masson Trichrome staining was performed with Masson Stain Kit (Solarbio, China) according to the manufacturer instructions. Collagen fibers were stained for blue and muscle fibers were stained for red.

Culture and identification of HUVECs

Primary HUVECs were purchased from ScienCell Research Laboratories (8000). Beginning at passage two, cells were characterized by immunofluorescence with specific antibodies against CD31 (1:100, Abcam, UK). HUVECs were cultured in endothelial cell medium (ECM; 1001, ScienCell Research Laboratories) containing 5% fetal bovine serum (FBS; 0025, ScienCell Research Laboratories), 1% endothelial cell growth supplement (ECGS, 1052, ScienCell Research Laboratories) and 1% antibiotic solution (P/S, 0503, ScienCell Research Laboratories). The cumulative cell population doubling level (CPDL) was calculated based on the following formula: $\Sigma PD = (\ln N1 - \ln N2)/\ln 2$, where N1 is the number of harvested cells and N2 is the number of seeded cells. Details were described in supplemental methods.

Lentiviral downregulation or upregulation of ANXA1 in HUVECs

HUVECs were seeded in 24-well plates (1×10^4 cells/well) and incubated overnight. At 20-30% confluence, the HUVECs were transduced with 2 μL of the sh-ANXA1 lentiviral vector (2×10^8 TU mL^{-1}) or 1 μL of the ANXA1 overexpression lentiviral vector (1×10^8 TU mL^{-1}) and 20 μL of enhanced infection solution (HitransG A, REVG004, Genechem, Shanghai) to downregulate or upregulate ANXA1 expression, respectively. Fresh medium was replaced 12 hours later. Forty-eight hours after infection, 1 μg mL^{-1} puromycin (S250J0, BasalMedia) was added to the culture medium, and the concentration was maintained at 0.5 μg/mL for at least 7 days for selection of stably transduced cells. The infection rate was determined by the observation of green

fluorescence in nearly 90% of the cells 72 hours after infection. Uninfected cells and empty lentiviral vector were used as controls. Cells from passage 2 to passage 8 were used for transduction.

Senescence-associated β-galactosidase staining

SA-β-gal staining was performed with a Senescence β-Galactosidase Staining Kit (C0602, Beyotime Institute of Biotechnology, China) according to the manufacturers' instructions.

Thoracic aortas were fixed with 4% paraformaldehyde overnight after harvesting. After washing twice with PBS, the aortas were dipped in fresh SA-β-gal staining solution and incubated at 37°C overnight. The aortas were then washed with PBS three times and photographed by a digital camera (Canon, Japan). The area percentage of positive staining was analyzed by ImageJ 1.51 software.

HUVECs seeded in 6-well plates were washed once with PBS (B320KJ, BasalMedia) to remove cell debris, fixed for 15 minutes in fixative solution and washed 3 times with PBS. The cells were then incubated with fresh SA-β-gal staining solution at 37°C for 12 hours. The percentage of senescent cells was determined under an inverted light microscope (TS100, Nikon, Japan) by random selection of 5 microscopic fields of view at 10× and 20× magnification and was then calculated by ImageJ 1.51 software.

Cell cycle analysis

The cell cycle in HUVECs was analyzed by flow cytometry in an Accuri C6 Plus instrument (BD Biosciences, China) with a cell cycle detection kit (KGA512, KeyGen BioTech, Jiangsu, China) according to the manufacturer's guidance. A total of 1×10^{6} cells were harvested and centrifuged for 5 minutes at 2000 rpm. Precooled PBS was used for resuspension. The cells were fixed with 70% alcohol at 4°C overnight after another centrifugation step. The cells were washed with cold PBS twice for 3 minutes each by centrifugation at 1000 rpm and filtered through a 200-mesh strainer. Fresh propidium iodide (PI) staining solution was added to the cells at 4°C for 30 minutes in the dark. A total of 1×10^{4} cells were selected at a rate of no more than 60 cells/minute. Data were analyzed by ModFit 4.1.5 software.

Wound healing assay

Six-well plates were marked at intervals of 0.5 cm on the opposite side to delineate the area to be photographed. HUVECs were seeded in the 6-well plates (5×10^{5} cells/well) and incubated overnight. A 200 μL pipette tip was used to generate a wound. After washing with PBS, the wound was randomly photographed in 5 areas under an inverted microscope at 4× magnification. Incubation at 37°C was continued, and the wound was photographed at 24 hours and 48 hours. ImageJ 1.51 software was used for analysis of the repopulated area.

Tube formation assay

Matrigel (Corning, USA) was thawed on ice for 2 hours, and all pipettes and plates were precooled before the experiments. Matrigel was diluted 1:1 with ECM and added carefully into a prerinsed 24-well plate (200 μL/well). After incubation for 2 hours at 37°C in an incubator, the Matrigel had solidified, and 45000 trypsinized HUVECs were then seeded onto the Matrigel. After 4-6 hours of incubation in a 37°C incubator, tube formation was observed by microscopy and analyzed by ImageJ 1.51 software by determining the number of tubes.

qRT–PCR

RNA was isolated from HUVECs with a TransZol Up Plus RNA Kit (ER501-01, TransGen Biotech, Beijing, China) following the manufacturers' instructions. Quantitative RNA was used for cDNA synthesis with cDNA Synthesis SuperMix (11141ES60, Yeasen, Shanghai, China). SYBR Green Master Mix (11202ES08, Yeasen, Shanghai, China) was applied to quantify the mRNA expression of *GAPDH, ANXA1, P53, P21, P16, VCAM-1, ICAM-1, IL*-6, *IL-1β, NF-κ B, Iκ B, SERPINE 1*, and *TGFβ 2*. The CT values were compared with that of *GAPDH* h using the following formula: Fold change=$2^{-\Delta\Delta ct}$, Δct=ct (ct value of the target gene)-ct (ct value of the reference gene) and ΔΔct=Δct (ct value of the experimental group)- Δct (ct value of the control group). The primers used to amplify all the genes listed above were designed and synthesized by Sangon Biotech Company (Shanghai, China).

Western blotting

Protein was extracted from HUVECs and mouse aortas with RIPA lysis buffer (P0013B, Beyotime Institute of Biotechnology, China) containing a protease & phosphatase inhibitor cocktail (P1050, Beyotime Institute of Biotechnology, China). An Enhanced BCA Protein Assay Kit (P10010S, Beyotime Institute of Biotechnology, China) was used for protein quantification (30 μg/lane) following the instructions. Using 10% or 12% SDS-polyacrylamide gels, proteins were separated by electrophoresis at a voltage of 120 V and transferred to polyvinylidene difluoride membranes (IPVH00010, Millipore Corporation, USA) at a constant current of 200 mA. The membranes were then blocked with 5% milk for 1 hour and incubated separately with primary antibodies. The primary antibodies were as follows: rabbit monoclonal anti-VCAM1 (1:1000, ab134047), mouse monoclonal anti-P21 (1:1000, ab80633), rabbit monoclonal anti-ICAM1 (1:1000, ab222736, ab109361), rabbit monoclonal anti-ANXA1 (1:2000, ab214486), rabbit polyclonal anti-P16 (1:1000, ab189034), purchased from Abcam (Cambridge, UK); and rabbit monoclonal anti-phospho-histone H2A.X (1:1000, 9718) and rabbit monoclonal anti-NF-κB (1:1000, 3034), rabbit monoclonal anti-CyclinE1(1:500,20808), rabbit monoclonal anti-Rb (9309),purchased from Cell Signaling Technology. Mouse monoclonal anti-β-Actin (1:10000), mouse monoclonal anti-flag (1:1000, F1804, Sigma), and mouse monoclonal anti-P53 (1:1000, 48818S, Cell Signaling Technology) antibodies were also purchased for experiments.

Library preparation for transcriptome sequencing

A total of 1 μg RNA per sample was used as input material for RNA sample preparation. Poly-T oligo-attached magnetic beads were used for purification of mRNA from total RNA. Fragmentation was carried out using divalent cations under elevated temperature conditions in First Strand Synthesis Reaction Buffer (5×). Random hexamer primers and M-MuLV Reverse Transcriptase (RNase H-) were used to synthesize first-strand cDNA. Second-strand cDNA synthesis was subsequently performed using DNA Polymerase I and RNase H. Remaining overhangs were converted into blunt ends via exonuclease/polymerase activity. After 3’ adenylation of DNA fragments, adaptors with a hairpin loop structure were ligated to prepare the fragments for hybridization. To preferentially select cDNA fragments with a length of 370~420 bp, the library fragments were purified with an AMPure XP System (Beckman Coulter, Beverly, USA). Then, PCR was performed with Phusion High-Fidelity DNA polymerase, Universal PCR Primers and Index (X) Primer. Finally, PCR products were purified (AMPure XP system), and the library quality was assessed on the Agilent Bioanalyzer 2100 system.
